# Assessment of Mental and Chronic Health Conditions as Determinants of Health Care Needs and Digital Innovations for Women With Sexual Dysfunction: Cross-Sectional Population-Based Survey Study in Germany

**DOI:** 10.2196/71301

**Published:** 2025-08-27

**Authors:** Selina Marie Kronthaler, Tatjana Tissen-Diabaté, Maria Margarete Karsten, Jens-Uwe Blohmer, Klaus Michael Beier, Laura Hatzler

**Affiliations:** 1 Institute of Sexology and Sexual Medicine, Charité – Universitätsmedizin Berlin, Charité Platz 1, Berlin, 10117, Germany, +49 30 450 617 139, +49 30 450 529 992; 2 Institute of Social Medicine, Epidemiology and Health Economics, Charité – Universitätsmedizin Berlin, Berlin, Germany; 3 Department of Gynecology and Breast Center, Charité – Universitätsmedizin Berlin, Berlin, Germany; 4 Department of Urology, Charité – Universitätsmedizin Berlin, Berlin, Germany

**Keywords:** sexual health, chronic health conditions, mental health, female sexual dysfunction, sexual pain disorders, sexual distress, diagnosis, *ICD-11*, network analysis, *International Classification of Diseases, 11th Revision*

## Abstract

**Background:**

A chronic health condition (CHC) is a recognized risk factor for experiencing problems in sexual function (PSF). According to the *International Classification of Diseases, 11th Revision* (*ICD-11*), the development of severe symptoms of sexual distress is the defining criterion for clinically relevant sexual dysfunction. Data on the contribution of specific CHCs to clinically relevant sexual dysfunction symptoms and related health care needs are limited, hindering targeted interventions.

**Objective:**

This study examines the prevalence of PSF, sexual dysfunction, and sexual distress; assesses associations with CHC status; evaluates sexual dysfunction diagnoses; and explores health care preferences.

**Methods:**

Data collection in this cross-sectional population-based survey study was based on a questionnaire developed with patient and public involvement and administered by YouGov to a representative sample of adults in Germany. Analyses included 1970 women with and without CHCs and different CHC subgroups (mental health–related, gynecological, cardiovascular and metabolic, infectious and inflammatory, cancer, pain-related, and neurological). The outcomes measured were PSF, clinically relevant sexual dysfunction symptoms, sexual distress (Female Sexual Distress Scale-Desire/Arousal/Orgasm [FSDS-DAO]), and self-reported sexual dysfunction diagnoses. Multivariable regression and network analysis explored associations among CHC subgroups, PSF, sexual dysfunction, and FSDS-DAO scores.

**Results:**

Among 1970 cisgender women (mean age 49.6, SD 16.0 years), 1186 (60.2%) reported CHCs. The 6-month PSF prevalence was 75.2% (820/1090) in women with CHCs and 62.5% (399/638) in women without CHCs. Clinically relevant sexual dysfunction symptoms were less prevalent (CHC: 202/1046, 19.3% vs no CHC: 68/601, 11.3%). Multivariable regression models showed an association between sexual dysfunction and CHCs (odds ratio [OR] 2.56, 95% CI 1.90‐3.49), which was the strongest for women with mental health–related CHCs (OR 2.31, 95% CI 1.70‐3.13) and cancer CHCs (OR 2.00, 95% CI 1.45‐2.78). Being in a relationship was a protective factor for clinically relevant distress among women with CHCs. Network analysis showed positive associations of PSF with gynecological and mental health–related CHCs and of sexual dysfunction with mental health–related, gynecological, and cancer CHCs. Women with sexual dysfunction symptoms reported low rates of sexual dysfunction diagnosis (CHC: 39/200, 19.4% vs no CHC: 6/55, 10.7%) and treatment (CHC: 16/146, 11.0% vs no CHC: 3/40, 7.0%). Gynecologists were the preferred health care providers for sexual dysfunction. The most commonly reported unmet need was a lack of information. Digital solutions, such as apps and websites with exercises, were desired as health care innovations.

**Conclusions:**

The burden of CHCs on women’s sexual health extends beyond functional sexual impairment, with high rates of clinically relevant sexual distress. Cancer and mental health conditions are the strongest predictors of sexual dysfunction. Despite the high prevalence of sexual dysfunction in women with CHCs, access to diagnosis and treatment is limited. Digital offerings could help address these unmet needs.

## Introduction

### Background

Problems in sexual function (PSF), including problems with sexual desire, arousal, orgasm, or pain during sex, are common among adult women. In Germany, the 12-month self-reported prevalence rate of these problems in the general female population is 45.7% [1]. According to the *International Classification of Diseases, 11th Revision* (*ICD-11*), the presence of distress associated with PSF is the defining criterion for the diagnosis of any sexual dysfunction and sexual pain disorder [2]. The 12-month prevalence of sexual dysfunction, including clinically relevant distress, is estimated at 16.5% in the general population [1]. Previous literature has identified several well-established biopsychosocial risk factors for sexual dysfunction. These include relationship-related issues, history of abuse, religious affiliation, poor physical or mental health, and chronic diseases [3-5]. Chronic health conditions (CHCs) affect more than 60% of the general population in Germany [6]. CHCs, along with treatment side effects and complications, often affect other physical functions beyond the specific impairments caused by the conditions themselves. The impact of the inability to participate in social activities can cause emotional distress and reduce quality of life [7-10]. The elevated rates of sexual complaints among women with CHCs, such as 66.0% in patients with cancer [11], 61.4% in patients with diabetes [12], and 61.4% in patients with inflammatory bowel disease [13], highlight the strong association between physical conditions and sexual dysfunction. Moreover, mental health conditions, such as affective disorders, have been shown to be associated with higher rates of sexual dysfunction [3-5,14]. Specifically, the rates of sexual dysfunction range from 45% to 95% in individuals with major depression and 33% to 75% in those with anxiety disorders, based on sexual functioning assessments [15]. The underlying causes are multifaceted and may include somatic changes such as altered blood flow, pelvic floor dysfunction, hormonal imbalances, neuropathy, and neurobiological factors [16-22]. Various explanatory models suggested that the high prevalence of sexual dysfunction in mental health conditions is driven by similar underlying cognitive and emotional processes, such as internalization and negative self-schema [23-26]. For instance, the symptoms of depression, such as lack of drive and reduced attention, can also manifest in sexual behavior [27]. Meta-analysis results derived from longitudinal studies have suggested the association between depression and sexual dysfunction to be bidirectional [28].

Despite the recognized importance of sexuality as a supporting resource for patients with CHCs, most studies and meta-analyses concerning the prevalence of sexual impairments among women with CHCs have relied on measures that assess impairments in sexual functioning irrespective of distress [11-13,15]. However, beyond its relevance for physical satisfaction and reproduction, sexuality serves as a source of attachment and intimacy [29], and thus comprises a fundamental aspect of well-being [30]. Therefore, clinically relevant symptoms of sexual dysfunction can have profound consequences for quality of life and relationships [14,31-35] and may elicit substantial sexual distress. While sexual distress serves as a recognized indicator of the clinical relevance of sexual dysfunction [2], it is frequently disregarded in studies on sexuality in patients with CHCs. Social relationships represent a well-established protective factor against mortality and morbidity [36,37]. Despite the recognized importance of sexuality as a supporting resource for patients with CHCs, studies that consider sexual distress as an indicator of the clinical relevance of sexual dysfunction symptoms remain scarce in this population.

Scientific societies emphasize the importance of tailoring multimodal treatments to the biopsychosocial factors involved in sexual dysfunction, including the assessment and treatment recommendations for sexual dysfunction [16,17,38]. Despite these guidelines and the prevailing need for information among patients, underdiagnosis and undertreatment of sexual dysfunction remain pervasive issues [39-44]. Contributing factors include limited time and insufficient training among health care providers (ie, psychotherapists, gynecologists, urologists, general practitioners, and nurses) to adequately address sexual health concerns. In response, scientific societies have implemented certified training curricula [45]. However, in Germany, as in many other countries, sex and couples therapy are often not reimbursed, leaving psychotherapy as the only reimbursed option for patients with sexual dysfunction [46,47]. Additionally, patients face barriers such as a lack of awareness regarding where to seek help [48] and shame in communicating needs related to sexual health concerns [49]. These barriers may explain why a plethora of studies have shown that patients prefer their health care providers to initiate discussions about sexuality [32,39]. The resulting obstacles to accessing treatment underscore the urgent need for research on necessary services and treatment goals to identify the most pressing unmet needs. Simultaneously, there is a growing preference for online information and treatment services [39,48,50,51], which have been shown to improve sexual function and reduce sexual distress in meta-analyses [52,53]. However, to meet the most urgent needs of women with CHCs and sexual dysfunction, these services must be carefully tailored to improve health care access.

### Objective

The aim of this study is to (1) assess and compare the prevalence of self-reported PSF, sexual dysfunction, and sexual distress among women without CHCs, among women with CHCs, and within CHC subgroups (mental health–related, gynecological, cardiovascular and metabolic, infectious and inflammatory, cancer, pain-related, and neurological conditions); (2) model the associations between sexual dysfunction and CHC status, including CHC subgroups; (3) evaluate the rates of diagnoses and compare them with self-reported sexual dysfunction symptoms; and (4) identify help-seeking behaviors and health care preferences among women with sexual dysfunction, based on their CHC status and the presence or absence of mental health conditions.

## Methods

### Study Design and Setting

This cross-sectional study used data from cisgender women drawn from a representative sample of the German population. The data collection was part of a research project funded by the patient and stakeholder engagement grant. Patient and public involvement (PPI) representatives were actively engaged in the development of a questionnaire on help-seeking behaviors and health care needs regarding sexual problems in Germany. This study followed the STROBE (Strengthening the Reporting of Observational Studies in Epidemiology) statement for cross-sectional studies [54] and the CHERRIES (Checklist for Reporting Results of Internet E-Surveys) [55] to ensure comprehensive and transparent reporting. The completed STROBE checklist is provided in Checklist 1.

### PPI Information

PPI participants comprised representatives advocating for topics or groups at risk for sexual health issues. The process adhered to PPI recommendations [56-58]. Representatives were required to have sufficient knowledge of the German language. Recruitment took place via email in July 2021. As there are no dedicated organizations for sexual dysfunction in Germany, major nonprofit organizations representing marginalized groups and others at increased risk of sexual dysfunction were approached and informed about the study. These included Vulvodynie Netzwerk, Verein Lichen Sclerosus, Deutsche Endometriose Vereinigung, PSSD Deutschland, Intergeschlechtliche Menschen e.V., and Aktionsbündnis Muslimischer Frauen, as well as key opinion leaders in the areas of female genital mutilation or cutting and sexual violence. Individuals interested in participating were encouraged to contact the respective organizations directly. After signing short-term contracts, 8 patient and public representatives with personal or professional experience in conditions, such as lichen sclerosus, vulvodynia, endometriosis, sexual violence, post-SSRI sexual dysfunction, female genital mutilation or cutting, sex development variations, and psychotherapy with Muslim women, were involved. Based on personal preference, 5 representatives joined the advisory group and 3 representatives served as co-researchers, with compensations of €150 (US $174) and €400 (US $464), respectively. PPI representatives were onboarded by imparting basic knowledge on sexual dysfunction (eg, etiology and treatment options) and the research methods. For all subsequent meetings, 2 internal researchers provided documents with background information and working materials and were available to answer questions at any time during the meetings. From July to December 2021, the co-researchers collaboratively developed and reviewed the questionnaire item set within 10 online meetings. The advisory group participated in 4 online sessions, making key contributions to decisions throughout the development process.

### Participants

Recruitment and data collection were conducted by YouGov Deutschland, an independent polling institute and research data and analytics group, from December 8 to 13, 2021. Participants had to be at least 18 years old and provide informed consent to YouGov in order to be eligible for participation. A random sample was drawn from the entire YouGov Germany panel of over 800,000 individuals. Sampling was conducted using YouGov’s proprietary “turbo sampling” method, which draws approximately 24 random subsamples per day across all active studies based on real-time completion rates within predefined quota cells (ie, age, sex, and federal state). These quotas are dynamically updated to ensure representativeness over the field period. Panel members received generic email invitations and were routed via a survey router to the most appropriate live survey based on their profile and current quotas, enabling efficient and unbiased quota fulfillment. To ensure data quality, participants with inconsistent responses to key demographics (eg, discrepancies between personal and household income) were excluded. Survey questions followed a fixed order. Participation was limited to 1 completion per person via a personal login to the survey and cookies. Statistical weights were applied to make the sample representative of the German adult population by age, sex, and federal state [59]. Panel members were recruited through advertising and website partnerships [60]. The survey link was provided to adult panelists until the sample size reached 4000 participants. The sample size was chosen based on practicality and feasibility considerations.

### Measures

#### Questionnaire Development

The questionnaire was developed through an iterative process in collaboration with PPI representatives. The final draft was submitted to YouGov, who provided feedback on item formulation, clarity, and technical implementation. To enhance clarity and feasibility, adjustments were made to the wording and length of items. YouGov also contributed recommendations regarding the use of filter questions. Based on their input, final adjustments were discussed and agreed upon jointly with the PPI representatives. Before launching the survey, internal and external pretests were conducted with 19 participants via YouGov. Survey items were typically presented 1 per page.

The finalized PPI-developed questionnaire, which required approximately 20 minutes to complete, comprised a total of 51 items for women. Of these, 23 items were newly developed through the PPI process, while 28 were selected from validated instruments assessing sexual distress (15 items), pain-related distress (1 item), and sexual dysfunction (8 items for women or 10 items for men), as detailed below. The Relationships Questionnaire-2 (RQ-2; 4 items) was also administered but has not been included in the present analyses [61]. Validated measures were initially proposed by the research team and subsequently reviewed and selected in collaboration with the PPI representatives. The PPI-developed items were organized into six thematic sections: (1) sociodemographic characteristics relevant for quota-based sampling (4 items; eg, sex, age, and federal state); (2) sexual health (2 items; eg, awareness for PSF and awareness for help); (3) self-reported received diagnosis (1 item; presence of CHC and sexual dysfunction diagnosis); (4) biopsychosocial protective and risk factors (2 items; eg, life events, general health status, and interpersonal experience); (5) help-seeking behavior (6 items; eg, source of information and received treatment); and (6) health care needs (8 items; eg, treatment goals and preferred offerings). The PSF awareness item in the sexual health section served as a filter for questions about past help-seeking behavior. In addition, 12 sociodemographic and health items (eg, religious affiliation and relationship status) from YouGov’s existing registration dataset were purchased and incorporated to align with recommendations on overall survey length. The full codebook is available in Multimedia Appendix 1.

#### Sociodemographic, Behavioral, and Sexual Factors

Total items regarding sociodemographic variables included age (in years), education level (>12 years), monthly net income, employment status, religious affiliation, relationship status, heterosexual orientation, migration background (ie, self or parental immigration after 1949), presence of ≥1 child in the household, history of pregnancy and child birth, current breastfeeding status, household size (≥2 individuals), responsibility for the majority of housework, primary caregiver status, and urban residence. Behavioral factors included current medication use, alcohol consumption (>1 drink per week), smoking status (ie, all respondents who did not select “I am a nonsmoker”), and low physical activity (ie, less than once per week). Sexual factors included experiences of sexual discrimination, masturbation, and partnered sexual activity within the past 12 months; a history of sexual trauma; and time spent in emotionally meaningful relationships. Operationalization and measurement details are provided in the codebook available in Multimedia Appendix 1.

#### *ICD-11* Screener for Sexual Dysfunction and Pain Disorders

Sexual dysfunction was assessed according to *ICD-11* using an early version of the Screening for Sexual Problems in Women (SSP-F) by Velten and Zarski [62], which included 8 questions. Four questions addressed the occurrence of PSF in the 4 domains of desire, arousal, orgasm, and pain on a 5-point Likert scale (1, not at all; 2, episodically; 3, sometimes; 4, often (75%); 5, always). If applicable, a further question assessed the related sexual distress level with the following response: not at all (1), a bit (2), partly (3), severe (4), or very severe (5). Sexual dysfunction was defined as a sexual problem in any of the 4 domains occurring at least episodically with severe or very severe distress, resulting in a binary sexual dysfunction variable (yes/no). PSF was defined as having any reported sexual problem, regardless of frequency or distress, resulting in a binary PSF variable (yes/no).

#### Female Sexual Distress Scale

The Female Sexual Distress Scale-Desire/Arousal/Orgasm (FSDS-DAO) from Derogatis et al [63] consists of 15 items capturing the level of distress associated with sexuality within the last 30 days on a 5-point Likert scale ranging from 0 (never) to 4 (always). The German translation was established according to the linguistic validation guidance by the Mapi Group [64]. A total sexual distress sum score was calculated, ranging from 0 to 60, with higher scores indicating greater sexual distress.

### Definition of CHC and Mental Health Status

Chronic conditions were grouped into mental health–related CHCs (MH; eg, depression, anxiety disorders, posttraumatic stress disorder, premenstrual dysphoric disorder, autism, and other mental health conditions), cardiovascular and metabolic CHCs (CV; eg, cardiovascular disease, atherosclerosis, hypertension, diabetes, dyslipidemia, and osteoporosis), gynecological CHCs (GY; eg, vulvodynia, vestibulodynia, lichen sclerosus, urinary tract problems, incontinence, endometriosis, dysmenorrhea, pelvic floor dysfunction, polycystic ovary syndrome, and infertility for more than 6 months), infectious and inflammatory CHCs (IN; eg, psoriasis, joint inflammation, rheumatoid arthritis, rheumatism, sexually transmitted infection, and HIV/AIDS), cancer CHCs (CA; eg, breast, cervical, uterine, vulvar, ovarian, and other cancers), pain-related CHCs (PA; eg, chronic pain, chronic pelvic pain, and bladder pain syndrome), and neurological CHCs (NE; eg, Alzheimer disease, dementia, epilepsy, multiple sclerosis, Parkinson disease, stroke, and cerebral palsy). Overall, participants were compared regarding the presence of CHCs (CHC vs no CHC). Among those with CHCs, an additional classification was made based on the presence of at least one MH among CHCs versus the presence of only somatic CHCs without any mental health conditions (MH+ vs CHC MH−).

### Definition of Comorbid and Distinct CHC Subgroups

The 7 CHC subgroups consisted of participants affected by at least one of the CHCs, ie, MH+, CV+, GY+, IN+, CA+, PA+, or NE+, referred to as “comorbid CHC” subgroups to highlight that women of these subgroups might not exclusively be affected by 1 CHC.

For the descriptive statistics of the prevalence estimates, participants with more than one CHC were excluded from the MH, CV, GY, IN, CA, PA, and NE subgroups. In analyses, subgroups excluding comorbid individuals have been referred to as “distinct CHC” subgroups to highlight that women in these subgroups are exclusively affected by a specific CHC.

### Efforts to Reduce Bias

Experts with personal or professional experience in PSF were included within the PPI process to reduce nonresponse bias and ensure the relevance of the study design.

### Statistical Methods

Statistical analyses were conducted using R, version 4.3.2, with the packages UpSetR [65] and IsingFit [66]. The dataset provided by YouGov included a weight variable aligning the data with the Microcensus 2014 regarding age, sex, and federal state. For descriptive analyses, weights were applied (weighted mean, SD, and frequency). Unweighted frequencies have been reported for main group sizes. A comorbidity analysis visualized the overlap of CHC subgroups. For descriptive analyses, such as the prevalence of PSF and sexual dysfunction, and the summary of FSDS-DAO, distinct subgroups were used. FSDS-DAO scores were compared between participants with and without CHCs, as well as across CHC groups, using linear regression models adjusted for age, sexual activity, and relationship status. Given the skewed distribution and discrete nature of FSDS-DAO as a summed score, a negative binomial regression model was additionally fitted as a sensitivity analysis to account for potential overdispersion and nonnormality of residuals. Multivariable logistic regression assessed the association between sexual dysfunction and CHCs, using (1) a binary variable for CHC status, (2) a binary variable for MH status, or (3) binary variables for comorbid CHC subgroups (MH+, CV+, GY+, IN+, CA+, PA+, and NE+), adjusting for age, sexual activity, and relationship status. Odds ratios (ORs) and 95% CIs have been reported. For ordinal variables with ≥4 categories, median and IQR were estimated. To explore the interrelationships among the CHC subgroups and 4 sexual dysfunction domains, a network analysis was conducted using the methodology proposed by Epskamp and Fried [67] for binary data. Therefore, the Ising model with regularized estimation nodewise logistic regression was applied using the IsingFit R package by van Borkulo et al [66], with the OR rule and an EBIC hyperparameter of *γ*=0.25. Interaction parameters (β), representing the strength of the interaction between 2 variables, and threshold parameters, indicating the probability of occurrence within the sample, have been reported. Results have been reported by subgroup, including CHC, no CHC, MH+, CHC MH−, and comorbid CHC subgroups, except for the descriptive statistics of the prevalence analysis, which used the distinct CHC subgroups. Missing values were not imputed, and “not specified” and “not answered” responses were treated as missing. All analyses were considered exploratory, with no significance level or adjustment for multiple comparisons.

### Ethical Considerations

This human participant study was approved by Charité – Universitätsmedizin Berlin (EA4/221/21). YouGov obtained informed consent from its panelists for data collection and transmitted pseudonymized data to Charité for analysis. Participants were compensated with 500 YouGov points (equivalent to €1 or US $1.16) for completing the questionnaire. Participants could opt out of any item and skip questions without providing a response.

## Results

### Characteristics of the Study Population

**Figure 1.**
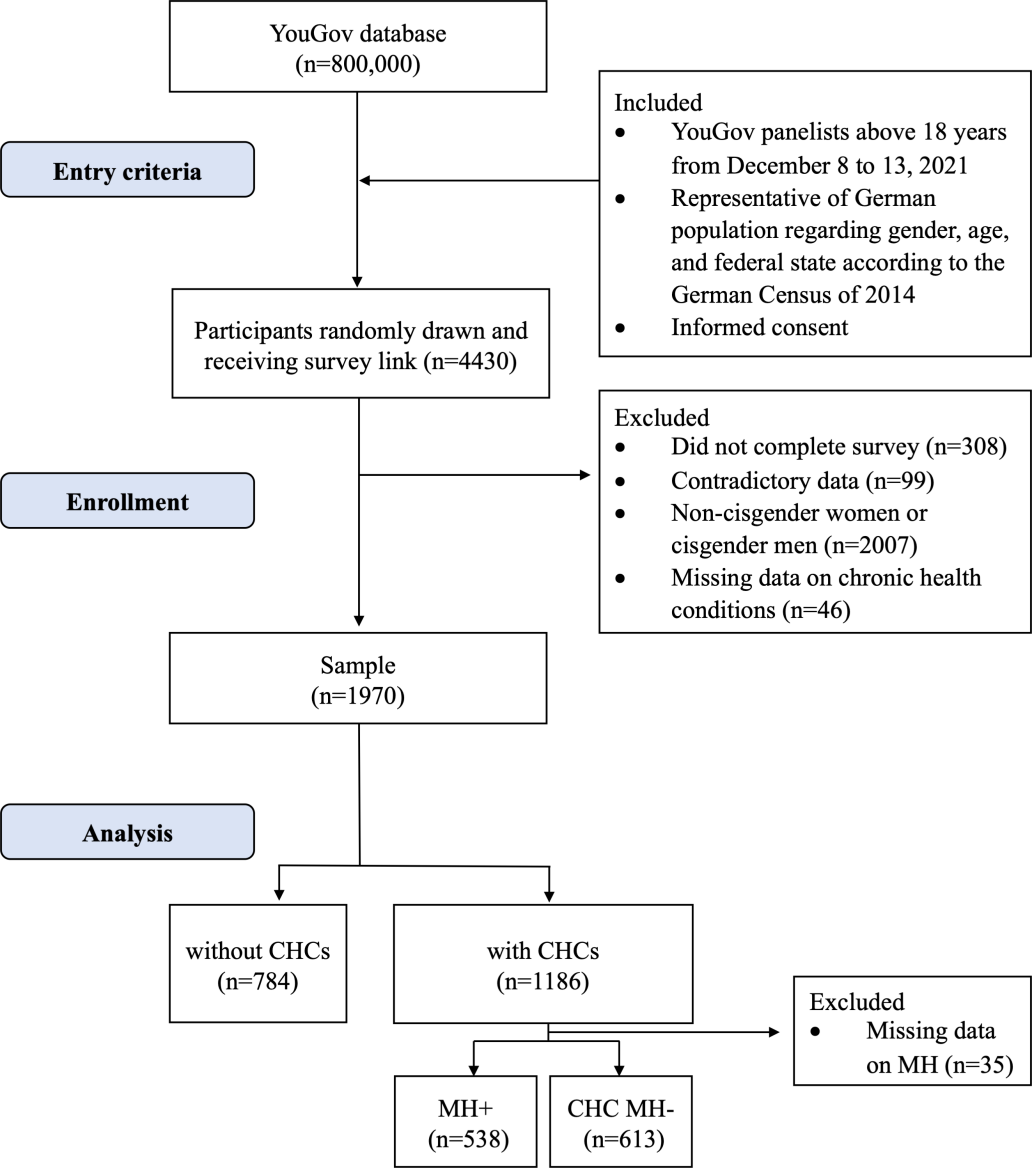
Flowchart of participants with unweighted numbers. CHC: chronic health condition; CHC MH−: chronic health conditions excluding mental health conditions; MH: mental health–related chronic health conditions; MH+: comorbid mental health–related chronic health conditions.

**Figure 2.**
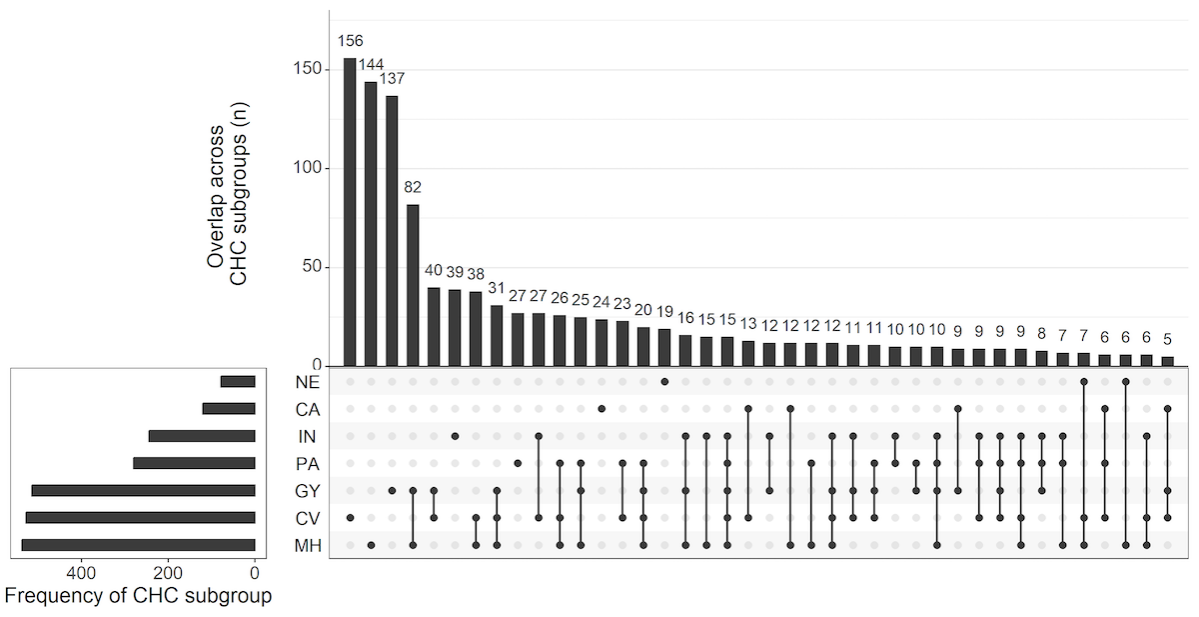
Intersection of chronic conditions visualized using an UpSet plot. The plot displays co-occurrence patterns among 7 chronic health conditions (CHCs). The left bar plot indicates the overall prevalence of each individual condition across the sample, regardless of whether it co-occurs with other conditions. The upper bar plot represents the frequency of specific intersection sets, showing how many individuals are affected by particular combinations of conditions. Each intersection set is denoted by connected dots in the matrix below the upper bar plot, where filled dots indicate the presence of a condition in the corresponding combination. This visualization facilitates the identification of common comorbidity patterns within the dataset. CA: cancer chronic health conditions; CV: cardiovascular and metabolic chronic health conditions; GY: gynecological chronic health conditions; IN: infectious and inflammatory chronic health conditions; MH: mental health–related chronic health conditions; NE: neurological chronic health conditions; PA: pain-related chronic health conditions.

Of 4430 panelists invited to participate, 4122 started and completed the survey, resulting in a 93.0% completion rate. Of those, 99 panelists (2.4%) were excluded due to contradictory demographic data, leaving a total of 4023 data records. After further exclusion of 2007 noneligible participants (unweighted: n_cis-men_=1787, n_noncis_=220) and 46 participants not responding to the question regarding CHCs, 1970 participants were included in the analysis (mean age 49.6, SD 16.0 years). For the flow of participants, see Figure 1. Women with CHCs but no MH (CHC MH−; 613/1935, 31.9%) were older than women with MH+ (538/1935, 27.8%), with mean ages of 54.3 (SD 15.5) years and 49.0 (SD 14.3) years, respectively (Table 1 and Table S1 in Multimedia Appendix 2 for CHC subgroups). Women with sexual dysfunction were younger than those without sexual dysfunction, both among those with CHCs (mean age: 46.1, SD 15.6 years vs 53.3, SD 14.2 years) and without CHCs (mean age: 32.8, SD 12.7 years vs 47.4, SD 15.8 years). Among all participants, the majority reported being in a relationship (1157/1929, 60.0%). In the last 12 months, 18.1% (331/1829) were sexually active with another person, and 34.3% (628/1829) were sexually active with themselves. Multiple CHCs were present in 32.6% (633/1939) of participants, with mental health and gynecological conditions being the most frequent comorbidities (Figure 2).

**Table 1.** Characteristics of the study population stratified by CHC^a^ and mental health–related CHC status (weighted frequencies; group sizes are unweighted).

Characteristic	No CHC (n=784)	CHC		
		All (n=1186)	MH+^b^ (n=538)	CHC MH−^c^ (n=613)
Age (years), mean (SD)	46.0 (16.6)	52.0 (15.1)	49.0 (14.3)	54.3 (15.5)
Age groups (years), n (%)				
18‐30	172 (22.4)	137 (11.7)	77 (14.6)	59 (9.8)
31‐40	147 (19.2)	137 (11.7)	67 (12.6)	70 (11.4)
41‐50	124 (16.1)	188 (16.0)	104 (19.7)	78 (12.8)
51‐65	213 (27.8)	484 (41.3)	228 (43.1)	237 (39.1)
>65	111 (14.5)	225 (19.2)	53 (10.1)	163 (26.9)
Education (>12 years), n (%)	348 (45.4)	469 (40.0)	227 (43.0)	231 (38.0)
Monthly net income (€), n (%)				
<2500 (US $2900)	494 (78.8)	854 (82.6)	402 (85.4)	430 (80.2)
2500‐5000 (US $2900-5800)	118 (18.9)	165 (16.0)	66 (13.9)	96 (17.8)
>5000 (US $5800)	15 (2.3)	14 (1.4)	3 (0.6)	11 (2.0)
Employed, n (%)	430 (62.1)	490 (43.9)	219 (44.3)	260 (44.3)
Religious, n (%)	437 (61.3)	674 (59.9)	278 (54.4)	376 (64.8)
Relationship, n (%)	461 (60.5)	696 (59.6)	295 (56.1)	380 (62.7)
Heterosexual, n (%)	662 (93.7)	1004 (91.2)	449 (89.3)	523 (92.5)
Migration background, n (%)	147 (19.6)	172 (14.7)	77 (14.6)	92 (15.2)
Children (≥1) in same household, n (%)	205 (26.7)	223 (19.0)	96 (18.3)	119 (19.5)
Pregnancy or child birth^d^, n (%)	33 (4.8)	62 (5.5)	24 (4.7)	37 (6.2)
Breastfeeding^d^, n (%)	23 (3.3)	17 (1.5)	8 (1.6)	9 (1.6)
Household size ≥2, n (%)	562 (73.2)	800 (68.3)	341 (64.6)	437 (71.9)
Majority of housework, n (%)	347 (50.8)	627 (55.7)	285 (55.8)	330 (55.8)
Primary caregiver, n (%)	88 (13.0)	146 (12.9)	63 (12.4)	77 (13.1)
Urban area, n (%)	290 (37.8)	475 (40.6)	229 (43.3)	229 (37.8)
Behavioral risk factors, n (%)				
Medication for CHCs	85 (12.2)	538 (47.3)	269 (52.0)	256 (43.1)
Alcohol consumption^e^	554 (80.2)	878 (77.2)	394 (76.1)	462 (77.8)
Smoking	345 (49.9)	567 (49.8)	262 (50.6)	289 (48.7)
Low physical activity^e^	525 (68.5)	779 (66.5)	347 (65.6)	401 (65.9)
Sexual discrimination	0 (0.0)	15 (1.3)	7 (1.4)	8 (1.3)
Sexual behavior, n (%)				
Masturbation^d^	422 (37.1)	206 (29.8)	231 (44.7)	186 (31.3)
Partnered sexual activity^d^	132 (19.0)	199 (17.5)	120 (23.3)	77 (13.0)
Sexual trauma^d^	18 (2.7)	78 (6.9)	54 (10.4)	24 (4.1)
Spending time in relationships^d^	284 (41.7)	484 (43.0)	193 (37.7)	283 (47.8)

a CHC: chronic health condition.

b MH+: comorbid mental health–related chronic health conditions.

c CHC MH−: chronic health conditions excluding mental health conditions.

d In the past 12 months.

e Less than once per week.

### Sample Representativeness and Comparison With Microcensus Data

The full unweighted sample from YouGov (n=4023) was compared with the 2014 official German Microcensus data [59]. Key demographic characteristics, including sex, age distribution, and federal state, showed comparable distributions (see Tables S3 and S4 in Multimedia Appendix 2).

### Prevalence of PSF and Sexual Dysfunction Symptoms and Sexual Distress in Distinct CHC Subgroups

#### Prevalence

**Figure 3.**
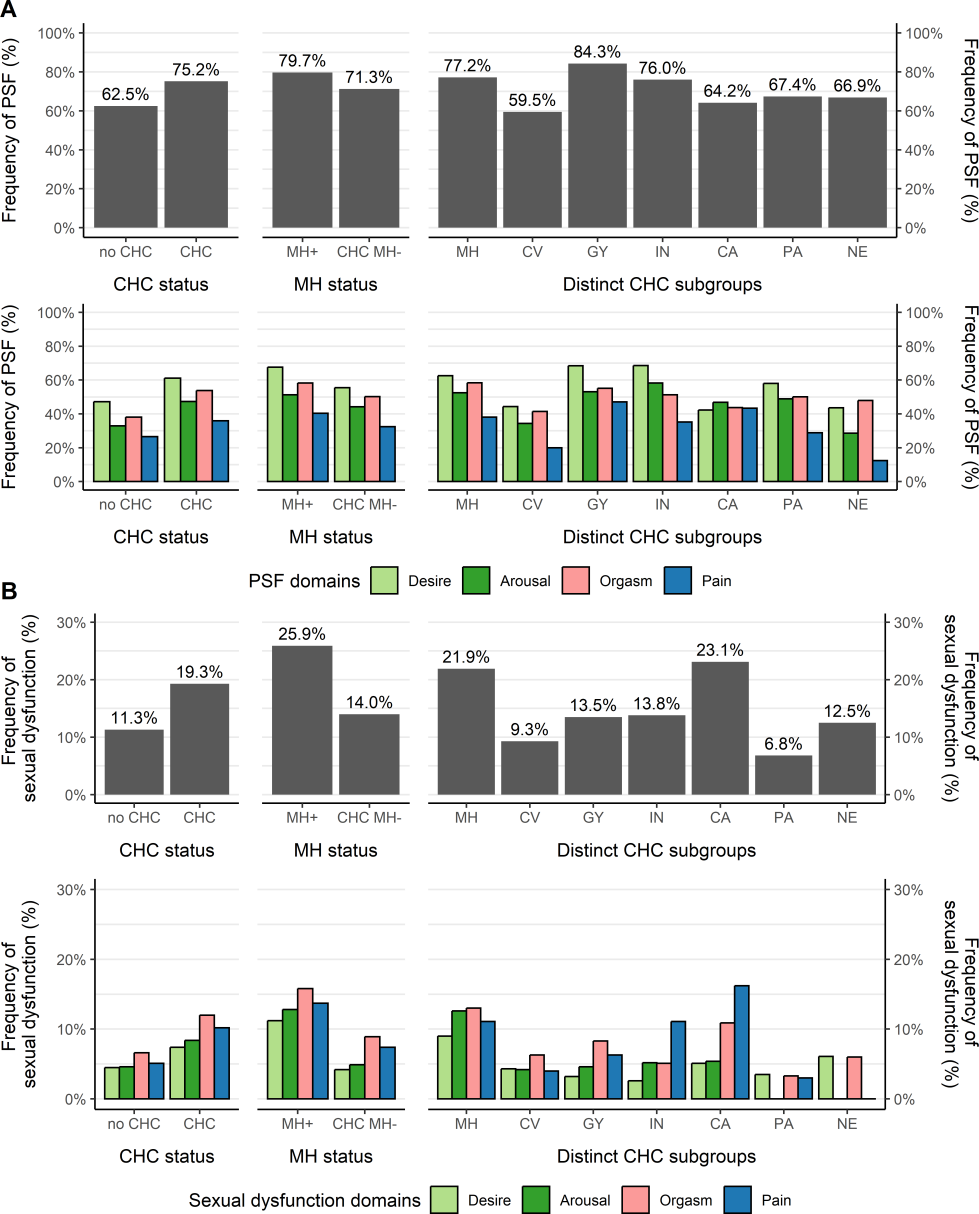
Prevalence of problems in sexual function (PSF) and sexual dysfunction. Panel A shows the prevalence of PSF stratified by overall chronic health condition (CHC) status, mental health–related CHC (MH) status, and distinct CHC subgroups. Panel B presents the corresponding prevalence of sexual dysfunction. For both PSF and sexual dysfunction, prevalence estimates are further broken down by individual PSF or sexual dysfunction domains. CA: cancer chronic health conditions; CHC MH−: chronic health conditions excluding mental health conditions; CV: cardiovascular and metabolic chronic health conditions; GY: gynecological chronic health conditions; IN: infectious and inflammatory chronic health conditions; MH+: comorbid mental health–related chronic health conditions; NE: neurological chronic health conditions; PA: pain-related chronic health conditions.

Overall, women with CHCs had a higher prevalence of PSF symptoms (CHC: 820/1090, 75.2% vs no CHC: 399/638, 62.5%; OR 1.82, 95% CI 1.48‐2.24) and sexual dysfunction symptoms with clinical relevance (CHC: 202/1046, 19.3% vs no CHC: 68/601, 11.3%; OR 1.87, 95% CI 1.40‐2.53) (Table 2). Low sexual desire was the most frequently experienced PSF in women with and without CHCs. In contrast, sexual dysfunction symptoms with clinical relevance were reported most frequently in the domain of orgasm, independent from CHC status, and in all distinct CHC subgroups, except for CA and IN, which most frequently had sexual pain disorders. For an illustration of PSF and sexual dysfunction prevalence, see Figure 3.

**Table 2.** Prevalence of problems in sexual function and sexual dysfunction, and descriptive summary of Female Sexual Distress Scale-Desire/Arousal/Orgasm findings by CHC^a^ status and distinct CHC subgroups (weighted frequencies; group sizes are unweighted).

Variable	No CHC (n=768)	CHC (n=1172)	OR^b^ (95% CI)	MH^c^ (n=144)	CV^d^ (n=156)	GY^e^ (n=137)	IN^f^ (n=39)	CA^g^ (n=24)	PA^h^ (n=27)	NE^i^ (n=19)
PSF^j^, n (%)										
≥Domain	399 (62.5)	820 (75.2)	1.82 (1.48‐2.24)	397 (79.7)	84 (59.5)	109 (84.3)	26 (76.0)	13 (64.2)	17 (67.4)	11 (66.9)
Desire	318 (47.2)	674 (61.1)	1.76 (1.45‐2.14)	82 (62.6)	64 (44.3)	89 (68.4)	24 (68.5)	9 (42.3)	14 (58.0)	7 (43.6)
Arousal	212 (32.9)	518 (47.3)	1.83 (1.50‐2.24)	68 (52.5)	49 (34.3)	69 (53.1)	20 (58.2)	10 (46.8)	12 (48.9)	5 (28.6)
Orgasm	240 (38.1)	582 (53.8)	1.90 (1.56‐2.31)	76 (58.4)	58 (41.4)	72 (55.1)	17 (51.3)	8 (43.7)	12 (50.1)	8 (47.9)
Pain	169 (26.6)	387 (35.9)	1.54 (1.25‐1.91)	50 (38.1)	28 (20.0)	61 (47.1)	12 (35.2)	8 (43.4)	7 (28.8)	2 (12.4)
Sexual dysfunction, n (%)										
≥Domain	68 (11.3)	202 (19.3)	1.87 (1.40‐2.53)	124 (25.9)	13 (9.3)	17 (13.5)	5 (13.8)	4 (23.1)	2 (6.8)	2 (12.5)
Desire	30 (4.5)	81 (7.4)	1.69 (1.11‐2.63)	12 (9.0)	6 (4.3)	4 (3.2)	1 (2.6)	1 (5.1)	1 (3.5)	1 (6.1)
Arousal	29 (4.6)	91 (8.4)	1.91 (1.26‐2.96)	16 (12.6)	6 (4.2)	6 (4.6)	2 (5.2)	1 (5.4)	0 (0)	0 (0)
Orgasm	42 (6.6)	129 (12.0)	1.91 (1.34‐2.77)	17 (13.0)	9 (6.3)	11 (8.3)	2 (5.1)	2 (10.9)	1 (3.3)	1 (6.0)
Pain	32 (5.1)	109 (10.2)	2.10 (1.42‐3.18)	14 (11.1)	6 (4.0)	8 (6.3)	4 (11.1)	3 (16.2)	1 (3.0)	0 (0)
FSDS-DAO**^k^** (score 0‐60), median (IQR)										
All	3 (0.0‐13.0)	9 (1.0‐21.0)	—^l^	13 (3-23)	4 (0‐11)	10 (4-19)	6 (1-19)	3 (3-23)	4 (1-14)	1 (0‐2)
Women with PSF	9 (2.0‐19.0)	14 (5.0‐25.0)	—	16 (7-25)	9 (3-16)	13 (5-21)	14 (4-24)	23 (3-25)	8 (3-17)	0 (0‐2)
FSDS-DAO (score 0‐60), mean (SD)										
All	8.4 (11.1)	12.9 (12.9)	—	14.7 (13.1)	7.8 (10.6)	12.3 (10.4)	10.6 (10.6)	12.3 (13.8)	8.1 (9.3)	3.2 (6.2)
Women with PSF	12.5 (12.3)	16.2 (13.2)	—	17.5 (13.1)	12.2 (11.9)	14.2 (10.4)	12.9 (10.5)	15.9 (15.3)	10.0 (9.4)	2.5 (5.1)

a CHC: chronic health condition.

b Odds ratios (ORs) are reported for the comparison of women with and those without chronic health conditions.

c MH: mental health–related chronic health conditions.

d CV: cardiovascular and metabolic chronic health conditions.

e GY: gynecological chronic health conditions.

f IN: infectious and inflammatory chronic health conditions.

g CA: cancer chronic health conditions.

h PA: pain-related chronic health conditions.

i NE: neurological chronic health conditions.

j PSF: problems in sexual function.

k FSDS-DAO: Female Sexual Distress Scale-Desire/Arousal/Orgasm.

l Not applicable.

#### Sexual Distress

**Figure 4.**
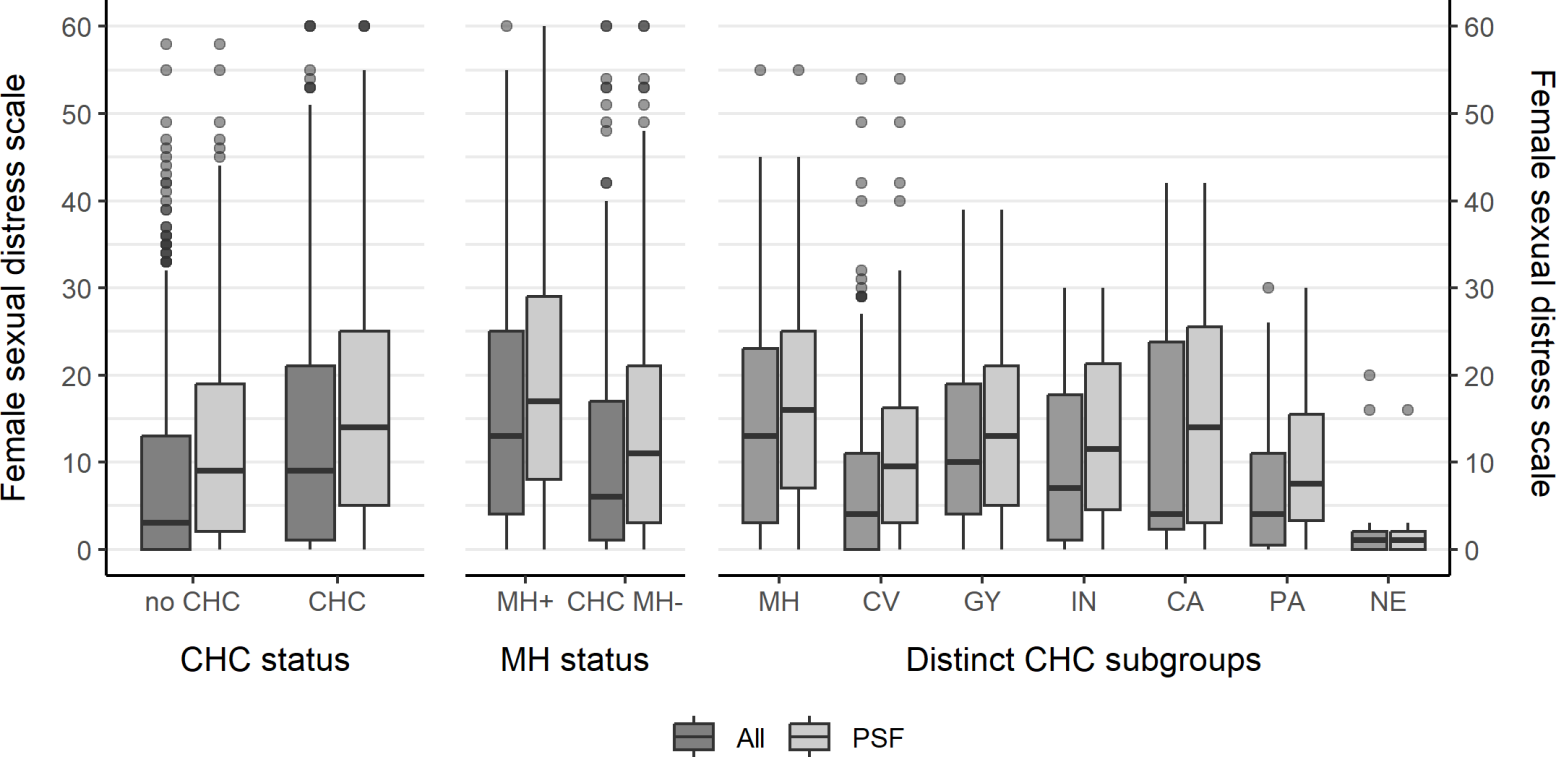
Sexual distress measured by the Female Sexual Distress Scale-Desire/Arousal/Orgasm by chronic health condition (CHC) status, mental health–related CHC (MH) status, and distinct CHC subgroups. CA: cancer chronic health conditions; CHC MH−: chronic health conditions excluding mental health conditions; CV: cardiovascular and metabolic chronic health conditions; GY: gynecological chronic health conditions; IN: infectious and inflammatory chronic health conditions; MH+: comorbid mental health–related chronic health conditions; NE: neurological chronic health conditions; PA: pain-related chronic health conditions; PSF: problems in sexual function.

As assessed by the FSDS-DAO, participants with CHCs reported higher sexual distress (mean 12.9, SD 12.9) than those without CHCs (mean 8.4, SD 11.1). Among women with CHCs, women with MH reported higher mean scores (mean 16.0, SD 13.6) than those without MH (mean 10.2, SD 11.6). Among participants with PSF, the highest FSDS-DAO scores were noted in women with cancer (n=11; median 23, IQR 3‐25) and those with MH (n=92; median 16, IQR 7‐25) (Figure 4).

### Association of CHC Status and CHC Subgroups With PSF, Sexual Dysfunction, and Sexual Distress

#### Multivariable Regression Models

For the analysis of a CHC as a risk factor for sexual dysfunction, logistic regression models were adjusted for age, sexual activity, and relationship status. Being sexually active was a protective factor, while being in a relationship increased the risk for sexual dysfunction in all models. Having any CHC was considerably associated with sexual dysfunction (adjusted OR 2.56, 95% CI 1.90‐3.49; *P*<.001) (see Model 1 in Table 3). Model 2 showed that the odds of sexual dysfunction symptoms were twice as high in the MH+ group compared to the CHC MH− group (adjusted OR 2.00, 95% CI 1.45‐2.78; *P*<.001). Model 3 revealed the strongest associations between CHCs and sexual dysfunction for participants with MH+ (adjusted OR 2.31, 95% CI 1.70‐3.13; *P*<.001) and those with cancer (adjusted OR 1.98, 95% CI 1.18‐3.25; *P*=.008). A subgroup analysis for the relationship status revealed that the association between CHCs and sexual dysfunction was lower in women who were in a relationship (OR 2.06) than in those who were not (OR 4.18) (Table S2 in Multimedia Appendix 2).

**Table 3.** Multivariable logistic regression models for sexual dysfunction using binary CHC^a^ status (model 1), mental health–related CHC (MH) status (model 2), and comorbid CHC subgroups (model 3).

Variable	Model 1 (n=1675)		Model 2 (n=1675)		Model 3 (n=1491)	
	OR^b^ (95% CI)	*P* value	OR (95% CI)	*P* value	OR (95% CI)	*P* value
CHC (yes)	2.56 (1.90‐3.49)	<.001	—^c^	—	—	—
Age	0.96 (0.95‐0.97)	<.001	0.97 (0.96‐0.98)	<.001	0.96 (0.95‐0.97)	<.001
Sexual activity^d^	0.90 (0.65‐1.25)	.55	0.79 (0.52‐1.18)	.25	0.80 (0.55‐1.15)	.24
Relationship	1.34 (1.01‐1.77)	.04	1.27 (0.92‐2.78)	.16	1.56 (1.15‐2.13)	.005
MH+^e^/CHC MH−^f^ (MH)	—	—	2.00 (1.45‐2.78)	<.001	—	—
CHC						
MH+	—	—	—	—	2.31 (1.70‐3.13)	<.001
CV+^g^	—	—	—	—	1.35 (0.95‐1.91)	.09
GY+^h^	—	—	—	—	1.40 (1.03‐1.90)	.03
IN+^i^	—	—	—	—	1.21 (0.85‐1.89)	.34
CA+^j^	—	—	—	—	1.98 (1.18‐3.25)	.008
PA+^k^	—	—	—	—	1.28 (0.85‐1.89)	.23
NE+^l^	—	—	—	—	1.16 (0.57‐2.19)	.67

a CHC: chronic health condition.

b OR: odds ratio.

c Not applicable.

d Partnered sexual activity in the last 12 months.

e MH+: comorbid mental health–related chronic health conditions.

f CHC MH−: chronic health conditions excluding mental health conditions.

g CV+: comorbid cardiovascular and metabolic chronic health conditions.

h GY+: comorbid gynecological chronic health conditions.

i IN+: comorbid infectious and inflammatory chronic health conditions.

j CA+: comorbid cancer chronic health conditions.

k PA+: comorbid pain-related chronic health conditions.

l NE+: comorbid neurological chronic health conditions.

For the analysis of a CHC as a risk factor for sexual distress, linear models revealed an average increase in the FSDS-DAO score by 6 points for CHC vs no CHC (Table S5 in Multimedia Appendix 2). In addition, a negative binomial regression was applied owing to 25% zero inflation and a poor fit with linear regression, confirming the presence of a CHC as a risk factor for sexual distress (data not shown).

#### Network Analyses

**Figure 5.**
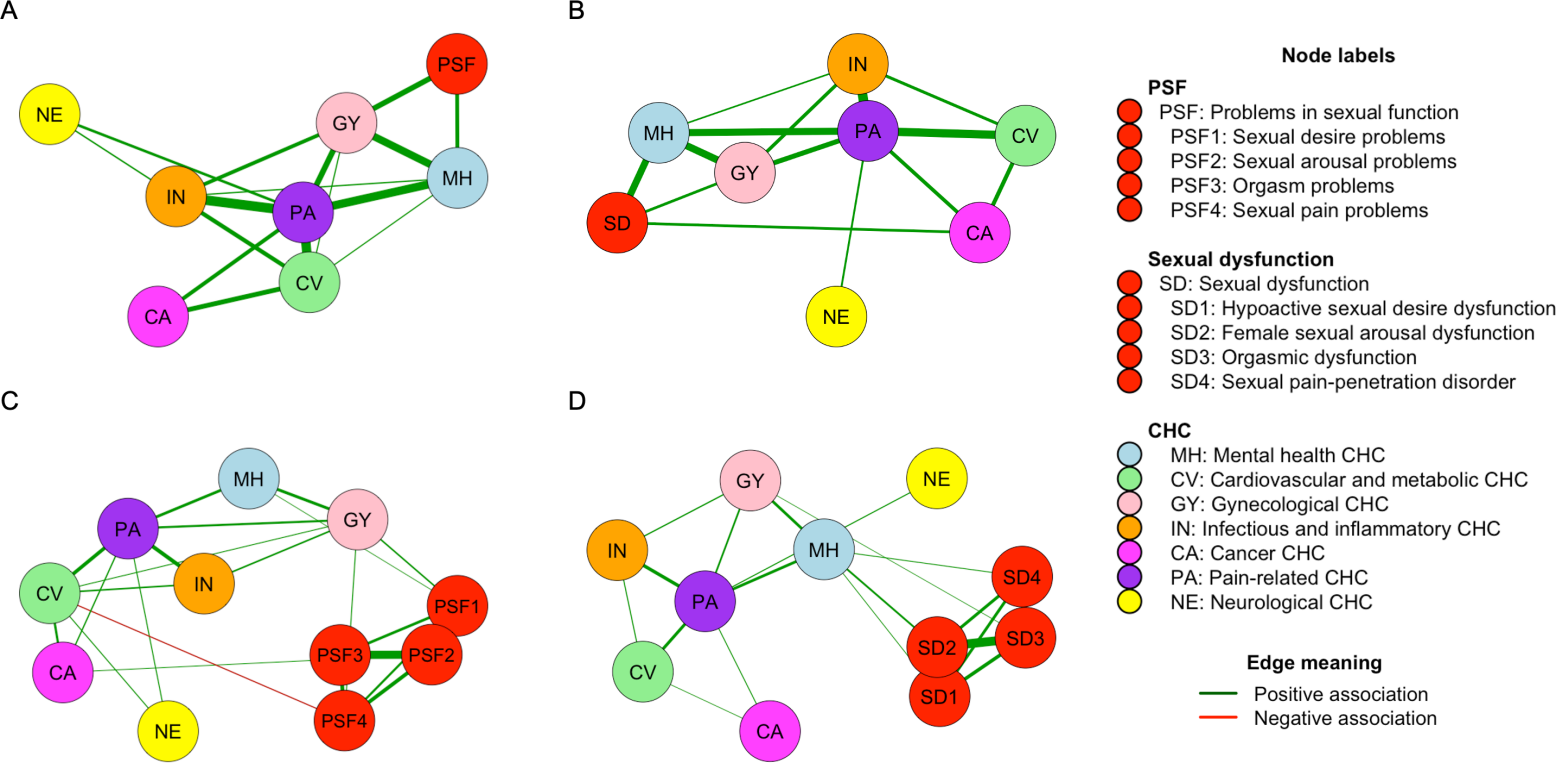
Network analyses of chronic health condition (CHC) subgroups and (A) total problems in sexual function (PSF), (B) total sexual dysfunction (SD), (C) individual domains of PSF, and (D) individual domains of SD. Nodes represent outcome variables, and edges represent interaction parameters (β), with thickness indicating the strength of the association.

The network analyses of the comorbidity structure of CHC subgroups with PSF and CHC subgroups with sexual dysfunction are presented visually in Figure 5. With a hyperparameter value of 0.25, associations were observed between PSF and GY (β_PSFGY_=.59) and between PSF and MH (β_PSFMH_=.42). The PSF domains showed strong intercorrelations, most pronounced between desire and arousal (β_PSF1PSF2_=2.45) and arousal and orgasm (β_PSF2PSF3_=2.45). Sexual dysfunction had moderate positive associations with MH (β_SDMH_=.75) and weak associations with GY (β_SDGY_=.32) and CA (β_SDCA_=.32). As with the PSF domains, the sexual dysfunction domains were also strongly intercorrelated. For further information on interaction parameter β, see Tables S6-S9 in Multimedia Appendix 2. Using the spring layout, PA was centrally located in all networks and showed high regression coefficients across different conditions, especially with IN and CV. CV had the highest thresholds in all networks, except for PSF in network A, indicating its high probability of presence (Table S10 in Multimedia Appendix 2).

#### Received Diagnoses in Women With Sexual Dysfunction

The prevalence of self-reported sexual dysfunction diagnoses in the female study population was 7.4% (129/1749) for any sexual dysfunction diagnosis, 4.8% (83/1750) for hypoactive sexual desire disorder, 2.0% (35/1750) for orgasmic disorder, and 1.7% (30/1749) for sexual pain-penetration disorder. When compared to the prevalence rates of clinically relevant sexual dysfunction symptoms according to *ICD-11*, substantial gaps were observed in all sexual dysfunction domains. Assessment of diagnosis rates among women reporting sexual dysfunction symptoms revealed large gaps between sexual dysfunction prevalence based on *ICD-11* criteria and actual diagnosis rates, with differences depending on CHC status and sexual dysfunction domains. Although underdiagnosis could be shown for all groups, higher rates of sexual dysfunction diagnosis were found among women with CHC (CHC: 39/200, 19.4% vs no CHC: 6/55, 10.7%; OR 2.00, 95% CI 0.85‐5.53), in particular among those with MH+ (MH+: 29/124, 23.5% vs CHC MH−: 10/76, 12.8%; OR 2.10, 95% CI 0.99‐4.81). However, within sexual dysfunction domains, higher diagnosis rates for women with CHCs were detected only for hypoactive sexual desire disorder (CHC: 23/200, 11.7% vs no CHC: 2/55, 3.6%; OR 3.54, 95% CI 1.01‐22.45) and sexual pain-penetration disorder (CHC: 10/200, 4.9% vs no CHC: 1/55, 1.6%; OR 3.11, 95% CI 0.55‐70.20). In contrast, women with CHCs had slightly lower rates of diagnosis when they had orgasmic disorder (CHC: 12/200, 6.2% vs no CHC: 4/55, 7.5%; OR 0.82, 95% CI 0.28‐2.94).

### Health Care Needs

#### Help-Seeking Behavior

The most reported primary sources of information about sexual problems were the internet (CHC: 63/150, 42.1% vs no CHC: 23/39, 59.7%; MH+: 45/102, 44.8% vs CHC MH−: 18/48, 37.2%) and gynecological visits (CHC: 61/150, 40.5% vs no CHC: 15/39, 38.0%; MH+: 39/102, 38.2% vs CHC MH−: 21/48, 44.2%). Access to therapy for women with sexual dysfunction was limited (CHC: 16/145, 11.0% vs no CHC: 2/39, 7.0%). The median duration to start treatment after symptom onset was shorter for women with CHCs (CHC: 3‐4 months vs no CHC: 5‐6 months). Access to psychotherapy was rare. However, it was slightly higher for women with CHC (CHC: 18/149, 11.8% vs no CHC: 1/38, 2.5%; OR 5.30, 95% CI 1.01‐106.69) but lower for those without MH (MH+: 14/101, 14.4% vs CHC MH−: 3/48, 6.6%). The most reported barriers for women with sexual dysfunction and CHCs were shame (CHC: 81/197, 41.1% vs no CHC: 28/65, 43.0%), fear of not being taken seriously (CHC: 56/197, 28.4% vs no CHC: 25/65, 38.3%), and a lack of information about who to contact (CHC: 54/197, 27.5% vs no CHC: 19/65, 28.8%). For complete data on the help-seeking behavior of women with sexual dysfunction, see Table S11 in Multimedia Appendix 2.

#### Preferred Access to Care and Treatment

Women with sexual dysfunction preferred gynecological visits for information, regardless of CHC status (CHC: 113/197, 57.4% vs no CHC: 42/66, 63.5%) and the presence or absence of MH (MH+: 71/120, 59.1% vs CHC MH−: 42/75, 56.1%). Favoring psychotherapy as a treatment for sexual dysfunction was only marginally different between CHC groups (CHC: 51/202, 25.5% vs no CHC: 14/68, 21.2%) but more frequent in women with MH (MH+: 42/124, 34.0% vs CHC MH−: 9/76, 12.4%). In contrast to women without CHC, more women with CHC preferred the sexual dysfunction treatment options of specialized clinics (CHC: 46/202, 22.6% vs no CHC: 9/68, 13.2%; OR 1.94, 95% CI 0.93‐4.45), drugs (CHC: 48/202, 23.9% vs no CHC: 6/68, 9.2%; OR 3.09, 95% CI 1.37‐8.15), and surgery (CHC: 14/202, 6.8% vs no CHC: 1/68, 1.5%; OR 4.71, 95% CI 0.95‐77.33). For women with sexual dysfunction and CHCs, the most important treatment goals were increases in body and sexual self-esteem (CHC: 82/196, 42.0%; MH+: 57/121, 46.9% vs CHC MH−: 26/73, 35.2%), relationship satisfaction (CHC: 76/196, 38.7%; MH+: 44/121, 36.6% vs CHC MH−: 31/73, 42.1%), and sexual satisfaction (CHC: 75/196, 38.5%; MH+: 46/121, 37.9% vs CHC MH−: 29/73, 39.4%). Regarding functional domains, improvement in desire was more often rated as important by women with MH (MH+: 43/121, 35.8% vs CHC MH−: 20/73, 27.6%) and pain by women without MH (MH+: 32/121, 26.4% vs CHC MH−: 24/73, 32.2%). For complete data on preferred access to care and treatment, see Table S12 in Multimedia Appendix 2.

#### Preferred Future Developments

Improved access to information was the most frequently desired development (60/179, 33.6%). Regarding the ratings of digital offers (10-point Likert scales), reimbursement by health insurance (mean 8.3, SD 2.4) and contact with sexual medicine experts (mean 7.5, SD 2.4) were considered most relevant by all women with sexual dysfunction. Women with sexual dysfunction and MH more often reported a need for direct contact per video call (MH+: 21/111, 19.0% vs CHC MH−: 5/70, 7.1%). For an overview of favored future developments and digital expert contact, see Table 4.

**Table 4.** Health care needs of women with sexual dysfunction by chronic health condition (CHC) and mental health–related CHC status.

Variable	Without CHC^a^ (n=69)	With CHC (n=207)	OR^b^ (95% CI)	MH+^c^ (n=127)	CHC MH−^d^ (n=78)
Favored future developments, n	59	179	—^e^	106	71
New drugs, n (%)	6 (10.2)	29 (16.2)	1.71 (0.72‐4.72)	13 (12.5)	16 (22.2)
New surgery, n (%)	3 (5.5)	15 (8.6)	1.60 (0.53‐6.53)	10 (9.2)	6 (7.9)
Better information, n (%)	12 (20.3)	60 (33.6)	1.99 (1.01‐4.14)	33 (30.8)	27 (37.8)
Digital offers, n (%)					
Apps	13 (21.9)	43 (23.9)	1.12 (0.57‐2.32)	24 (22.5)	19 (26.8)
Websites	9 (16.0)	41 (23.2)	1.59 (0.76‐3.61)	27 (25.4)	15 (20.4)
Home aids	10 (16.9)	39 (21.7)	1.37 (0.66‐3.06)	22 (20.6)	16 (22.2)
With physical face-to-face treatments	5 (9.2)	20 (11.2)	1.25 (0.49‐3.69)	12 (11.0)	8 (11.7)
Contact with experts	11 (18.1)	38 (21.0)	1.20 (0.59‐2.64)	26 (24.7)	12 (16.2)
Training					
Physicians	10 (16.3)	29 (16.3)	1.00 (0.47‐2.30)	17 (16.3)	12 (16.9)
Psychologists	16 (27.7)	32 (17.8)	0.57 (0.29‐1.13)	25 (23.4)	7 (10.1)
Diversity and trauma^f^	5 (8.6)	29 (16.5)	2.09 (0.84‐6.25)	18 (17.2)	11 (15.9)
Expert contact in digital offers, n	61	183	—	111	70
Medical experts, n (%)	27 (45.2)	107 (58.5)	1.71 (0.96‐3.06)	69 (62.4)	37 (52.8)
Psychological experts, n (%)	21 (35.2)	90 (49.1)	1.77 (0.98‐3.25)	62 (56.3)	27 (39.2)
Video call, n (%)	13 (21.2)	27 (14.9)	0.65 (0.32‐1.39)	21 (19.0)	5 (7.1)
Chat, n (%)	18 (29.1)	56 (30.5)	1.07 (0.58‐2.04)	40 (36.5)	15 (20.7)
Email feedback, n (%)	14 (23.3)	46 (24.9)	1.09 (0.56‐2.20)	26 (23.6)	19 (27.7)

a CHC: chronic health condition.

b OR: odds ratio. ORs are reported for the comparison of women with and those without chronic health conditions.

c MH+: comorbid mental health–related chronic health conditions.

d CHC MH−: chronic health conditions excluding mental health conditions.

e Not applicable.

f Sensitivity training, for example, culture, religion, trauma, gender identity, and sexual orientation.

## Discussion

### Principal Findings

The objectives of this study were to assess and compare the prevalence of PSF, sexual dysfunction, and sexual distress among women with and without CHCs and in CHC subgroups, to model associations between sexual dysfunction and CHC subgroups, evaluate self-reported diagnosis rates versus self-reported symptoms, and identify help-seeking behaviors associated with CHCs and mental health status. Beyond confirming that the prevalence of sexual dysfunction is higher in women with CHCs compared to those without CHCs when applying *ICD-11* criteria, this representative study provides valuable evidence on the extent to which specific CHC subgroups are affected by the burden of sexual dysfunction. The prevalence of sexual dysfunction in women with MH or CA was twice that in women without CHCs. Network analyses revealed positive associations for PSF with GY and MH and for sexual dysfunction with CA. Notably, specific disorders, such as hypoactive sexual desire disorder, female sexual arousal dysfunction, and sexual pain-penetration disorder, were associated with MH, whereas orgasmic disorder was associated with GY. Although the prevalence of diagnosed sexual dysfunction was generally low among those with a positive *ICD-11* screening, women with MH had a higher prevalence of diagnosed sexual dysfunction compared to women with CHCs, excluding MH. Women with CHCs had higher odds of receiving a diagnosis of sexual dysfunction for hypoactive sexual desire disorder and sexual pain-penetration disorder, but not for orgasmic disorder. Previous help-seeking behavior had mainly occurred online and through gynecological visits, with low therapy initiation rates in all subgroups. In terms of health care needs, women with sexual dysfunction mostly preferred gynecological visits. Women with CHCs sought treatment goals related to body and sexual self-esteem, while those without CHCs prioritized sexual and relationship satisfaction. Most women indicated their interest in better information and digital health services, especially apps with information and exercises, with reimbursement being an important aspect for digital solutions. These findings allow for more accurate quantitative estimates of the need for sexual dysfunction interventions in women’s health care.

### Sexual Dysfunction Prevalence and Associations With CHCs

The sexual dysfunction prevalence of 16.4% for the general female population in our data is consistent with previous representative surveys in Germany reporting a prevalence of 16.5% [1]. Additionally, the frequency of experienced PSF in women with CHCs (59.5%‐84.3%) in our study sample is comparable to the findings in other studies, which have primarily used functional assessments to detect sexual dysfunction [11-13,15]. Besides applying functional measures, research on sexual health in patients with CHCs is often limited to specific groups of certain disciplines or conditions [3-5,27,32], giving rise to a gap in the literature regarding differences in prevalence rates of PSF and sexual dysfunction in women with and without CHCs and women with different CHCs. The discrepancy between PSF and sexual dysfunction rates, as well as FSDS-DAO scores, suggests that the presence of PSF does not necessarily imply clinically relevant distress. However, some CHCs seem to be more potent than others in increasing vulnerability for the development of sexual distress. In particular, while 5 out of 6 women (84%) with only GY experienced PSF, only a small proportion (13.5%) met the criteria for sexual dysfunction. In contrast, women with only CA had the second lowest prevalence of reported PSF (64%) but the highest prevalence of sexual dysfunction (23.4%). Women with only MH reported high prevalences of PSF (77.2%) and sexual dysfunction (21.9%) compared to all other subgroups.

Consistent with the highest prevalence of sexual dysfunction in our sample, the highest levels of sexual distress were reported by women with a history of only CA and MH, with median FSDS-DAO scores of 14.7 and 12.3, respectively.

In line with these findings, the network with sexual dysfunction and CHC subgroups found positive associations of sexual dysfunction with MH, GY, and CA. This was partly reflected in the multiple logistic regression analysis, which showed that a CHC was a risk factor for reporting sexual dysfunction in this population. In women with CHCs, those with MH showed stronger associations with sexual dysfunction than women with physical conditions, and the strongest associations for sexual dysfunction were with CA and MH. The strong links of MH with sexual dysfunction might be explained by similar underlying cognitive and emotional factors, such as internalizing behaviors, as discussed previously by Forbes et al [15,23-28]. Patients with cancer have also been shown to experience high levels of distress when faced with a life-threatening disease, which may also increase vulnerability to sexual distress [10,11,32]. This study highlights that female cancer survivors with sexual dysfunction face the most severe impact on their sexual health. The high rate of reported willingness to pay substantial amounts for effective therapy by women with cancer further supports this conclusion. In contrast, women with gynecological conditions often have questions about sexual health, but only a few are willing to pursue sex therapy [44]. This suggests a high need for information, but not necessarily for therapy, which is consistent with the lower prevalence of distress in our data. Overall, PSF may primarily reflect questions and informational needs, whereas meeting sexual dysfunction criteria may indicate a need for a targeted therapeutic intervention.

Interestingly, there was a difference in the frequency of symptoms regarding domains of sexual function. Low sexual desire was the most common sexual problem experienced, but orgasmic disorder was the most prevalent sexual dysfunction for all groups, except women with inflammatory conditions and cancer.

Partnered women with CHCs had a reduced risk of sexual dysfunction compared to single women. Partnered women may benefit from more support and understanding from their partners, which may mitigate the impact of CHCs on sexual dysfunction. Studies have provided conflicting results about whether being in a relationship is a risk or protective factor for sexual dysfunction [4]. Relationship quality has been suggested as a mediator for the effect of relationships on sexual dysfunction [4,5,34]. However, bidirectional links need to be considered, as sexual dysfunction has been shown to be a risk factor for relationship conflict [35]. Our data indicate that being in a relationship is a risk factor for sexual dysfunction, but this may be due to detection bias.

### Health Care Preference

Our study found that the internet was the most commonly reported source for information help-seeking, which, on the one hand, underscores the importance of online resources in enhancing access to evidence-based information and treatment, as claimed by previous studies [50,52]. On the other hand, challenging barriers, such as low awareness of sexuality as a health issue, need to be addressed to link women to appropriate treatment effectively. The most important motivations for seeking treatment among all women with sexual dysfunction were to enhance body and sexual self-esteem and improve relationship and sexual satisfaction. This underpins the importance of focusing sexual health interventions on reframing the meaning of sexuality rather than solely targeting sexual functioning.

### Challenges in Health Care

The present data show that only a minority of women with sexual dysfunction received therapy (CHC: 10% vs no CHC: 7%). Previous studies have consistently highlighted a lack of treatment [39-44], a trend consistent with our findings of low diagnosis rates. Notably, our figures are lower than those reported in other recent cross-sectional studies in Germany, such as the study by Velten and Margraf [48], which reported a treatment rate of 47.7% for women. This discrepancy may stem from nonrepresentative study samples, likely drawing participants with greater awareness of sexual health concerns (ie, greater health literacy). Furthermore, this study aimed to examine diagnosis rates as an indicator of how aware health care providers are of sexual health as a medical need for women. We found a large gap between the symptoms of sexual dysfunction and the diagnoses received, with 82.4% of sexual dysfunction cases remaining undiagnosed. This would result in a falsely low sexual dysfunction diagnosis prevalence of 7.4% in the general population (CHC: 10.5% vs no CHC: 1.7%).

Gynecologists, identified as the preferred source for information and dialog partners in our study, have also been reported as the first point of contact in previous studies [48]. In our data, women without MH had lower rates of diagnosis and access to treatment than women with sexual dysfunction and MH. Despite this, sexual health concerns are still rarely recognized by health care providers. In outpatient psychotherapy clinics, sexual dysfunction diagnosis rates are as low as 0.2%‐1.2% [43], which contrasts sharply with the 25.9% prevalence of sexual dysfunction symptoms among women with only MH in our study. In line with these findings, women with MH in our study particularly highlighted the need for better training for psychologists. While MH, along with cancer, may carry the highest risk of developing sexual dysfunction, that is, clinically relevant sexual distress requiring treatment, the unmet medical need for sexual health support may be even greater among women without mental health issues.

Furthermore, our data indicate that reimbursement greatly affects therapy access for women with sexual dysfunction in Germany. Most women are willing to pay only small sums, which is insufficient for effective evidence-based interventions, with only 19.7% willing to pay more than €300 (US $348). This highlights the potential impact of a lack of reimbursement options for sex therapy and may explain why initiatives for nationally accredited sexual medicine training may reach only a small proportion of women with sexual dysfunction. Short-term psychotherapy (12 sessions) costs about €1200 (US $1392) [46]. Studies on the efficacy of treatments for sexual dysfunction usually suggest a reduced number of sessions compared to psychotherapy [47]. Assuming that of the 35.7 million adult women in Germany, about 5 million (14%) have sexual dysfunction but no MH, and given that 94.5% have not received therapy for sexual dysfunction and only psychotherapy is reimbursed, the socioeconomic burden would be €5.67 billion (US $6.59 billion) [46,68-70].

### Implications for Health Care and Research

The differences in priorities between groups support the need for tailored solutions to address individuals’ specific needs, as recommended by scientific societies [16,17,38]. Health care providers with certified training could play a critical role in addressing the significant gap in sexual health care for women with sexual dysfunction. However, it is important to address the limited time and training available to health care providers in this area, particularly among physicians, who may face greater challenges than psychotherapists [41-43]. Given the high prevalence of mental health problems among patients, addressing the needs and reimbursement challenges of those without MH is also critical. In addition, preventive programs that meet reimbursement criteria within the German health care system could provide valuable opportunities to improve relationships and promote sexuality as a resource, particularly for women with CHCs. These programs should also include single women to ensure that their specific needs are addressed.

### Quality of Representative Data

Comparison with the 2014 German Microcensus data indicated that the sample was representative of the selected criteria, including age, sex, and federal state [59]. This was reflected in the prevalence of CHCs, which was 60.0% in our study compared to 62.1% reported previously [6]. The completion rate was high (93.0%) relative to other sexuality studies [71]. However, descriptive characteristics revealed that the sample does not fully represent the general German population (eg, the smoking proportion) [72], indicating selection bias or limitations in item design and visibility. Additionally, certain CHCs may have been underreported despite a comprehensive item list, as indicated by non-CHC participants reporting CHC-related medication use. These factors may have contributed to minor deviations in sexual dysfunction prevalence estimates.

### Limitations

Our study has several limitations that should be considered. First, we observed notable demographic differences between women with and those without CHCs. Women with CHCs were generally older, less likely to be employed, and more likely to be retired, leading to disparities in monthly net household income. Additionally, the sexual dysfunction status was unknown for approximately 15% of participants due to missing data in the *ICD-11* screener. The diagnosis of female sexual arousal dysfunction could not be reported in this study, which might lead to an underestimation of sexual dysfunction prevalence. Furthermore, the data are based on a German and German-speaking survey sample, which limits the generalizability of our findings to other German subpopulations.

### Conclusions

The contribution of CHCs to the risk of sexual dysfunction appears to vary among different CHCs, with CA and MH showing the strongest association. The finding of limited access to sexual dysfunction diagnosis and treatment supports the contention of previous research that women’s sexual health is neglected in the health care system. The data also suggest that gaps in care are unevenly distributed across different CHCs. Women with only physical CHCs, particularly those with cancer, appear to be most affected by gaps in care. The interest in digital solutions, the need for reimbursement, or the specific needs of different target groups can serve as a basis for tailoring future health care innovations for women’s sexual health.

## Supplementary material

10.2196/71301 Multimedia Appendix 1 Codebook.

10.2196/71301 Multimedia Appendix 2 Supplementary data including study population characteristics, multivariable logistic regression models, weighted adjacency matrices, threshold parameters, and more.

10.2196/71301 Checklist 1 STROBE checklist.

## Abbreviations

CA: cancer chronic health conditions
CHC: chronic health condition
CHC MH−: chronic health conditions excluding mental health conditions
CV: cardiovascular and metabolic chronic health conditions
FSDS-DAO: Female Sexual Distress Scale-Desire/Arousal/Orgasm
GY: gynecological chronic health conditions
*ICD-11*: *International Classification of Diseases, 11th Revision*
IN: infectious and inflammatory chronic health conditions
MH: mental health–related chronic health conditions
NE: neurological chronic health conditions
OR: odds ratio
PA: pain-related chronic health conditions
PPI: patient and public involvement
PSF: problems in sexual function
STROBE: Strengthening the Reporting of Observational Studies in Epidemiology
