# Supplementary material for: Assessment of Mental and Chronic Health Conditions as Determinants of Health Care Needs and Digital Innovations for Women With Sexual Dysfunction: Cross-Sectional Population-Based Survey Study in Germany
Source: J Particip Med. 2025 Aug 27;17:e71301. doi: 10.2196/71301 (PMC12386550; doi:10.2196/71301)
Supplement: Multimedia Appendix 1 [file jopm-v17-e71301-s001.pdf]

OM_FuT_Charite_Frauengesundheit_2112

Version: 79

#Question display logic:
if (ask_age or ask_gender or ask_region)
#PAGE 6

Question type: ***Pdl***
#Question display logic:
if ask_gender and updated

[gender] Bitte geben Sie Ihr Geschlecht an.

<1>	männlich
<2>	weiblich

Question type: ***Pdl***
#Question display logic:
if ask_age and updated

[birthmonth] In welchem Monat sind Sie geboren? Bitte geben Sie Ihren Geburtsmonat als Zahl zwischen 1 und 12 ein.

Question type: ***Pdl***
#Question display logic:
if ask_age and updated

[birthyear] In welchem Jahr sind Sie geboren?

Question type: ***Pdl***
#Question display logic:
if ask_region and updated

[sta] In welchem Bundesland leben Sie?

<8>	Baden-Württemberg
<9>	Bayern
<11>	Berlin
<12>	Brandenburg
<4>	Bremen
<2>	Hamburg
<6>	Hessen
<13>	Mecklenburg-Vorpommern
<3>	Niedersachsen
<5>	Nordrhein-Westfalen
<7>	Rheinland-Pfalz
<10>	Saarland
<14>	Sachsen
<15>	Sachsen-Anhalt
<1>	Schleswig-Holstein
<16>	Thüringen
<17>	nicht in Deutschland

#Question display logic:
if panman.is_panelist or Gtype=="test"
#PAGE 8

*Question type: **Text***

Eine für Sie passende Umfrage steht nun bereit.

$subiect

Thema:

ca. $loi Minuten

Dauer:

$points

Punkte:

Bitte bedenken Sie, dass die voraussichtliche Dauer ein Durchschnittswert ist. Die Befragung kann bei Ihnen kürzer als auch länger dauern. Bei Fragen zur Umfrage wenden Sie sich bitte an panel@yougov.de. Viel Spaß bei der Beantwortung der folgenden Fragen.

#PAGE 9

Base: Alle Befragten
*Question type: **Multiple***
#SPD Category: sex

[ch00] Haben Sie schon einmal unter **sexuellen Problemen** gelitten, z. B. vermindertes sexuelles Verlangen, Probleme mit sexueller Erregung oder Erektion, Orgasmusprobleme, Schmerzen oder Schwierigkeiten beim Sex, Probleme mit frühzeitigem Samenerguss?

<1>	Ja, in der Vergangenheit
<2>	Ja, aktuell
<3 xor>	Nein
<977 fixed xor>	Weiß nicht / keine Angabe

#Question display logic:
if ch00.has_any([1,2])
#PAGE 10

Base: Personen, die aktuell oder in der Vergangenheit sexuelle Probleme hatten
Question type: ***Multiple***
#row order: randomize(rand_ch)
#SPD Category: sex

[ch01] Wo haben Sie im Fall von sexuellen Problemen bereits nach Informationen gesucht?
(Bitte wählen Sie alle zutreffenden Antwortmöglichkeiten aus.)

<3>	Partner / Partnerin
<4>	Freunde / Freundinnen
<5>	Hausarzt / Hausärztin
<6 if gender in ([2])>	Frauenarzt / Frauenärztin
<7>	Urologe / Urologin
<8>	Internet
<9>	Ratgeber (Literatur, Zeitschriften)
<10>	Netzwerk von Betroffenen (Chatforen, Selbsthilfegruppen)
<955 fixed>	Sonstiges
<966 fixed xor>	Ich habe noch nie nach Informationen gesucht.
<977 fixed xor>	Weiß nicht / keine Angabe

#Question display logic:
if ch00.has_any([1,2])
#PAGE 11

Base: Personen, die aktuell oder in der Vergangenheit sexuelle Probleme hatten
Question type: ***Multiple***
#row order: randomize(rand12)
#SPD Category: sex

[ch02] Mit wem haben Sie im Fall von sexuellen Problemen bereits gesprochen?
(Bitte wählen Sie alle zutreffenden Antwortmöglichkeiten aus.)

<3>	Hausarzt / Hausärztin	<11>	Familie
<4 if gender in ([2])>	Frauenarzt / Frauenärztin	<12>	Selbsthilfegruppe
<5>	Urologe / Urologin	<13>	Polizei
<6>	Psychiater / Psychiaterin	<14>	Rechtsanwalt / -anwältin
<7>	Anderer Arzt /andere Ärztin	<955 fixed>	Mit einer anderen Person
<8>	Psycholog:in / Psychotherapeut:in	<966 fixed xor>	Mit niemandem
<9>	Partner / Partnerin	<977 fixed xor>	Weiß nicht / keine Angabe
<10>	Freunde / Bekannte		

#PAGE 12

Base: Alle Befragten
Question type: ***Multiple***
#row order: randomize(rand_ch)

[ch03a] Angenommen, Sie hätten sexuelle Probleme und würden sich dadurch stark belastet fühlen.
Wo würden Sie sich am liebsten über sexuelle Probleme, die Sie stark belasten, informieren?
(Bitte wählen Sie alle zutreffenden Antwortmöglichkeiten aus.)

<3>	Partner / Partnerin
<4>	Freunde / Freundinnen
<5>	Hausarzt / Hausärztin
<6 if gender in ([2])>	Frauenarzt / Frauenärztin
<7>	Urologe / Urologin
<8>	Internet
<9>	Ratgeber (Literatur, Zeitschriften)
<10>	Netzwerk von Betroffenen (Chatforen, Selbsthilfegruppen)
<955 fixed>	Sonstiges
<966 fixed xor>	Gar nicht
<977 fixed xor>	Weiß nicht / keine Angabe

#PAGE 13

Base: Alle Befragten
*Question type: **Multiple***
#row order: randomize(rand12)

[ch04a] Angenommen, Sie hätten sexuelle Probleme und würden sich dadurch stark belastet fühlen.
Mit wem würden Sie am liebsten im Fall von sexuellen Problemen sprechen?
(Bitte wählen Sie alle zutreffenden Antwortmöglichkeiten aus.)

<3>	Hausarzt / Hausärztin	<11>	Familie
<4 if gender in ([2])>	Frauenarzt / Frauenärztin	<12>	Selbsthilfegruppe
<5>	Urologe / Urologin	<13>	Polizei
<6>	Psychiater / Psychiaterin	<14>	Rechtsanwalt / -anwältin
<7>	Anderer Arzt /andere Ärztin	<955 fixed>	Mit einer anderen Person
<8>	Psycholog:in / Psychotherapeut:in	<966 fixed xor>	Ich würde mit niemanden darüber sprechen.
<9>	Partner / Partnerin	<977 fixed xor>	Weiß nicht / keine Angabe
<10>	Freunde / Bekannte		

#PAGE 14

Base: Alle Befragten
*Question type: **Grid***
#row order: randomize
#SPD Category: sex

[ch05a] Inwiefern treffen die folgenden Aussagen auf Sie zu?

-[ch05a_1]	Ich habe das Gefühl, dass ich (mehr) Informationen in Bezug auf meine Sexualität benötige.
-[ch05a_2]	Ich habe das Gefühl, dass ich Hilfe in Bezug auf meine Sexualität benötige.
<1>	Trifft voll zu
<2>	Trifft teilweise zu
<3>	Trifft eher nicht zu
<4>	Trifft gar nicht zu
<977>	Weiß nicht / keine Angabe

#PAGE 15

Base: Alle Befragten
*Question type: **Multiple***
#row order: randomize

[ch07] Angenommen, Sie hätten sexuelle Probleme und würden sich dadurch stark belastet fühlen.
Was sind oder wären für Sie Hindernisse, im Gesundheitssystem Angebote für sexuelle Probleme, die Sie stark belasten, in Anspruch zu nehmen?
(Bitte wählen Sie alle zutreffenden Antwortmöglichkeiten aus.)

<2>	Fehlende Klarheit über Ansprechpersonen	<11>	Fehlende Angebote in meiner Region oder lange Wartezeiten
<3>	Fehlende Informationen über Behandlungsmöglichkeiten	<12>	Fehlende Angebote mit Berücksichtigung von Kultur und Religion
<4>	Unsicherheit über Wirksamkeit von Behandlungen	<13>	Fehlende Angebote mit Berücksichtigung von psychischen Problemen
<5>	Scham	<14>	Fehlende Angebote mit Berücksichtigung von körperlichen Erkrankungen
<6>	Kein Gesprächsbedarf	<15>	Fehlende Angebote mit Berücksichtigung meiner sexuelleren Orientierung und / oder Geschlechtsidentität
<7>	Angst, dass jemand davon erfährt	<955 fixed>	Sonstiges
<8>	Angst vor schwierigen Themen	<966 fixed xor>	Es gäbe keine Hindernisse
<9>	Angst, nicht ernst genommen zu werden	<977 fixed xor>	Weiß nicht / keine Angabe
<10>	Fehlende Zeit		

#Question display logic:
if ch00.has_any([1,2])
#PAGE 16

Base: Personen, die aktuell oder in der Vergangenheit sexuelle Probleme hatten
*Question type: **Multiple***
#SPD Category: sex

[ch08] Welche der folgenden Leistungen haben Sie aufgrund von sexuellen Problemen bereits in Anspruch genommen?
(Bitte wählen Sie alle zutreffenden Antwortmöglichkeiten aus.)

<1>	Steigerung der sportlichen Aktivität	<14 if gender in ([1])>	Penispumpe
<2>	Diät	<15>	Transkutane elektrische Nervenstimulation (TENS)
<3>	Psychotherapie	<16>	Operationen
<4 if gender in ([2])>	Angebote bei Frauenärztin / Frauenarzt	<17>	Sexual- und Paartherapie
<5 if gender in ([1])>	Angebote bei Urologin / Urologen	<18>	Selbsthilfegruppen
<6>	Angebote bei anderen Ärzten / Ärztinnen	<19>	Entspannungsverfahren (z.B. progressive Muskelrelaxation, Meditation)
<7 if gender in ([1])>	Medikamente (z. B. Viagra)	<20>	Physiotherapie
<8>	Hormontherapie	<21>	Biofeedback
<9>	Lokale Hormontherapie (Creme)	<22>	Körperbasierte Verfahren (z.B. Körperpsychotherapie, Massagen, Osteopathie)
<10>	Andere Medikamente	<955 fixed>	Sonstiges
<11>	Hilfsmittel für Beckenbodentraining	<966 fixed xor>	Ich habe keine Leistungen aufgrund von sexuellen Problemen in Anspruch genommen.
<12 if gender in ([2])>	Dilatoren (Dehnungsstifte)	<977 fixed xor>	Weiß nicht / keine Angabe
<13 if gender in ([2])>	Gleitgel		

#PAGE 17

Base: Alle Befragten
*Question type: **Multiple***
#row order: randomize

[ch09] Angenommen, Sie hätten sexuelle Probleme und würden sich dadurch stark belastet fühlen.
Welche existierenden Angebote würden Sie im Fall von sexuellen Problemen, die Sie stark belasten, am liebsten in Anspruch nehmen, wenn sie vor Ort und als Leistung der Krankenkassen zur Verfügung stünden?
(Bitte wählen Sie alle zutreffenden Antwortmöglichkeiten aus.)

<1>	Sprechstunde in spezialisiertem Zentrum	<11>	Übungsgeräte für Beckenbodentraining
<2>	Psychotherapie	<12 if gender in ([2])>	Dilatoren
<3>	Medikamente	<13>	Entspannungsverfahren

<4>	Operationen	<14 if gender in ([2])>	Vibratoren
<5>	Sexual- und Paartherapie	<15 if gender in ([1])>	Penispumpe
<6>	Austausch mit anderen Betroffenen (Peer Beratung, Selbsthilfegruppen)	<16>	Osteopathie und Massage
<7>	Biofeedback zur Wahrnehmung und Kontrolle von Köpervorgängen	<955 fixed>	Sonstige
<8>	Physiotherapie	<966 fixed xor>	Keine
<9>	Diät	<977 fixed xor>	Weiß nicht / keine Angabe
<10>	Sport		

#PAGE 18

Base: Alle Befragten
Question type: ***Multiple***
#row order: randomize #max number of choices: 3

[ch10] Welche neuen Entwicklungen im Gesundheitssystem hinsichtlich Hilfe bei sexuellen Problemen finden Sie am wichtigsten?
(Bitte wählen Sie bis zu drei Antwortmöglichkeiten aus.)

<1>	App mit Übungen und Informationen	<8>	Schulungen zum sensiblen Umgang mit Kultur, Religion, Trauma, geschlechtlicher Identität oder sexueller Orientierung u.a. Themen im Gesundheitswesen
<2>	Website mit Übungen und Informationen	<9>	Bessere Informationsangebote
<3>	Digitale Angebote in Verbindung mit körperlichen Verfahren in der Praxis (z. B. Physiotherapie)	<10>	Neue operative Eingriffe
<4>	Digitale Angebote in Verbindung mit Hilfsmitteln für Anwendung zu Hause	<11>	Neue Medikamente
<5>	Digitale Angebote mit Kontakt zu Expert:innen	<955 fixed>	Sonstiges
<6>	Schulungen in Sexualtherapie von Ärzt:innen	<966 fixed xor>	Keine
<7>	Schulungen in Sexualtherapie von Psycholog:innen	<977 fixed xor>	Weiß nicht / keine Angabe

#Question display logic:
if ch00.has_any([1,2])
#PAGE 19

Base: Personen, die aktuell oder in der Vergangenheit sexuelle Probleme hatten
Question type: ***Single***
#SPD Category: sex

[ch11] Haben Sie schon einmal eine Therapie aufgrund sexueller Probleme in Anspruch genommen?

<1>	Ja
<2>	Nein
<977 fixed xor>	Weiß nicht / keine Angabe

#Question display logic:
if ch11 in ([1])
#PAGE 20

Base: Personen, die Therapie wegen sexueller Probleme hatten
Question type: ***Single***
#SPD Category: sex

[ch11a] Wie lange hat es nach dem Symptombeginn der sexuellen Probleme gedauert, bis Sie eine darauf gezielte Therapie erhalten haben?

<1>	Unter 1 Monat
<2>	1 - 2 Monate
<3>	3 - 4 Monate
<4>	5 - 6 Monate
<5>	Länger als 6 Monate
<977 fixed xor>	Weiß nicht / keine Angabe

#PAGE 21

Base: Alle Befragten
Question type: ***Grid***
#row order: randomize

[ch12] Angenommen, Sie hätten sexuelle Probleme und würden sich dadurch stark belastet fühlen.
Wie wichtig wären Ihnen die folgenden Punkte bei der Anwendung von neuen **digitalen Therapieangeboten (App, Website)** für sexuelle Probleme, die Sie stark belasten?
(Bitte wählen Sie in jeder Zeile eine Antwortmöglichkeit aus)

-[ch12_1]	Integration von Behandlungsangeboten von verschiedenen Ärzt:innen in digitale Angebote (z.B. Frauenärzt:innen / Urolog:innen)
-[ch12_2]	Integration von Behandlungsangeboten von Psychotherapeut:innen in digitale Angebote
-[ch12_3]	Alleinstehendes digitales Angebot (mit oder ohne Kontakt zu Expert:innen)
-[ch12_4]	Möglichkeit des Kontakts zu Expert:innen für sexuelle Probleme
-[ch12_5]	Abrechnung über die Krankenkasse
-[ch12_6]	Anwendung zusammen mit Partner:in
<1>	Gar nicht wichtig<br>1
<2>	2
<3>	3
<4>	4
<5>	5
<6>	6
<7>	7
<8>	8
<9>	9
<10>	Sehr wichtig<br>10

<977> Weiß nicht / keine Angabe

#PAGE 22

Base: Alle Befragten
*Question type: **Multiple***
#row order: randomize

[ch13] Angenommen, Sie hätten sexuelle Probleme und würden sich dadurch stark belastet fühlen.
Was wäre Ihnen für den **Kontakt mit Expert:innen bei der Anwendung von neuen digitalen Therapieangeboten** für sexuelle Probleme, die Sie stark belasten, wichtig?
(Bitte wählen Sie alle zutreffenden Antwortmöglichkeiten aus.)

<2>	Termine zum Austausch per Chat
<3>	Termine zum Austausch per Video
<4>	Feedback per Mail
<5>	Kontakt zu sexualtherapeutischen Expert:innen aus der Gynäkologie (oder andere Ärzt:innen)
<6>	Kontakt zu sexualtherapeutischen Expert:innen aus der Psychotherapie
<955 fixed>	Sonstiges
<966 fixed xor>	Nichts
<977 fixed xor>	Weiß nicht / keine Angabe

#PAGE 23

*Question type: **Text***

Viele Menschen erleben gelegentlich Schwierigkeiten oder Probleme mit der sexuellen Funktion. Wie Menschen mit solchen Schwierigkeiten umgehen und wie sie sie erleben, ist sehr unterschiedlich. Diese können einmalig, phasenweise oder dauerhaft auftreten.
Die folgenden Fragen beziehen sich darauf, wie häufig solche Schwierigkeiten bei Ihnen in den letzten sechs Monaten aufgetreten sind und ob Sie darunter leiden.

#PAGE 24

Base: Alle Befragten
*Question type: **Single***
#SPD Category: sex

[ch14] Wie häufig haben Sie in den letzten sechs Monaten einen Mangel oder Verlust an sexuellem Verlangen erlebt?

<1>	Gar nicht
<2>	Selten
<3>	Manchmal
<4>	Meistens (75 %)
<5>	Immer / fast immer

#Question display logic:
if ch14 in ([2,3,4,5])
#PAGE 25

Base: Befragte, die Mangel oder Verlust an sexuellem Verlangen erlebt haben
Question type: ***Single***
#SPD Category: sex

[ch15] Wie sehr leiden Sie unter diesem Problem?

<1>	Gar nicht
<2>	Etwas
<3>	Teils, teils
<4>	Ziemlich
<5>	Sehr

#Question display logic:
if gender in ([2])
#PAGE 26

Base: Alle befragten Frauen
Question type: ***Single***
#SPD Category: sex

[ch16] Wie häufig haben Sie in den letzten sechs Monaten Probleme mit fehlender oder verminderter sexueller Erregung während sexueller Aktivitäten erlebt?

<1>	Gar nicht
<2>	Selten
<3>	Manchmal
<4>	Meistens (75 %)
<5>	Immer / fast immer

#Question display logic:
if gender in ([1])
#PAGE 27

Base: Alle befragten Männer
Question type: ***Single***
#SPD Category: sex

[ch17] Wie häufig haben Sie in den letzten sechs Monaten Schwierigkeiten erlebt, eine Erektion zu bekommen oder aufrechtzuerhalten?

<1>	Gar nicht
<2>	Selten
<3>	Manchmal
<4>	Meistens (75 %)
<5>	Immer / fast immer

#Question display logic:
if ch16 in ([2,3,4,5]) or ch17 in ([2,3,4,5])
#PAGE 28

Base: Befragte, die Schwierigkeiten hatten
Question type: ***Single***
#SPD Category: sex

[ch18] Wie sehr leiden Sie unter diesem Problem?

<1>	Gar nicht
<2>	Etwas
<3>	Teils, teils
<4>	Ziemlich
<5>	Sehr

#Question display logic:
if gender in ([2])
#PAGE 29

Base: Alle befragten Frauen
Question type: ***Single***
#SPD Category: sex

[ch19] Wie häufig haben Sie in den letzten sechs Monaten Schwierigkeiten gehabt, zum Orgasmus zu kommen oder haben eine deutlich verminderte Intensität des Orgasmus erlebt?

<1>	Gar nicht
<2>	Selten
<3>	Manchmal
<4>	Meistens (75 %)
<5>	Immer / fast immer

#Question display logic:
if gender in ([1])
#PAGE 30

Base: Alle befragten Männer
Question type: ***Single***
#SPD Category: sex

[ch20] Wie häufig haben Sie in den letzten sechs Monaten Schwierigkeiten mit einer deutlich verzögerten oder ausbleibenden Ejakulation gehabt?

<1>	Gar nicht
<2>	Selten
<3>	Manchmal
<4>	Meistens (75 %)
<5>	Immer / fast immer

#Question display logic:
if ch19 in ([2,3,4,5]) or ch20 in ([2,3,4,5])
#PAGE 31

Base: Befragte, die Schwierigkeiten hatten
Question type: ***Single***
#SPD Category: sex

[ch20a] Wie sehr leiden Sie unter diesem Problem?

<1>	Gar nicht
<2>	Etwas
<3>	Teils, teils
<4>	Ziemlich
<5>	Sehr

#Question display logic:
if gender in ([2])
#PAGE 32

Base: Alle befragten Frauen
Question type: ***Single***
#SPD Category: sex

[ch21] Wie häufig haben Sie in den letzten sechs Monaten Schmerzen beim Sex erlebt oder Schwierigkeiten beim Einführen z. B. eines Penis in die Vagina gehabt?

<1>	Gar nicht
<2>	Selten
<3>	Manchmal
<4>	Meistens (75 %)
<5>	Immer / fast immer

#Question display logic:
if gender in ([1])
#PAGE 33

Base: Alle befragten Männer
Question type: ***Single***
#SPD Category: sex

[ch22] Wie häufig haben Sie in den letzten sechs Monaten Probleme damit gehabt, bei sexueller Aktivität zu früh zu ejakulieren bzw. zu früh zum Orgasmus zu kommen? Die Häufigkeit bezieht sich hier auf die Anzahl der sexuellen Situationen, in denen das Problem auftrat.

<1>	Gar nicht
<2>	Selten
<3>	Manchmal
<4>	Meistens (75 %)
<5>	Immer / fast immer

#Question display logic:
if ch21 in ([2,3,4,5]) or ch22 in ([2,3,4,5])
#PAGE 34

Base: Befragte, die Schwierigkeiten hatten
*Question type: **Single***
#SPD Category: sex

[ch23] Wie sehr leiden Sie unter diesem Problem?

<1> Gar nicht
<2> Etwas
<3> Teils, teils
<4> Ziemlich
<5> Sehr

#PAGE 35

Base: Alle Befragten
*Question type: **Single***
#SPD Category: sex

[ch24] Wie häufig leiden Sie unter Schmerzen in der Region des äußeren Genitals, die spontan oder bei Berührungen (z. B. im Sitzen, beim Fahrradfahren oder beim Sex) auftreten?

<1> Gar nicht
<2> Selten
<3> Manchmal
<4> Meistens (75 %)
<5> Immer / fast immer

#Question display logic:
if ch24 in ([2,3,4,5])
#PAGE 36

Base: Befragte, die Schmerzen hatten
*Question type: **Single***
#SPD Category: sex

[ch24a] Wie sehr leiden Sie unter diesem Problem?

<1> Gar nicht
<2> Etwas
<3> Teils, teils
<4> Ziemlich
<5> Sehr

#PAGE 37

Base: Alle Befragten
*Question type: **Multiple***
#row order: randomize

[ch25] Angenommen, Sie hätten sexuelle Probleme und würden sich dadurch stark belastet fühlen.
Was wären für Sie die **wichtigsten Ziele**, die durch eine Behandlung von sexuellen Problemen, die Sie stark belasten, erreicht werden sollten?
(Bitte wählen Sie bis zu drei Antwortmöglichkeiten aus.)

<1>	Mehr Orgasmen	<10>	Mehr sexuelle Erregung
<2>	Höhere Lebensqualität	<11>	Mehr Wissen über Sexualität
<3>	Weniger oder keine Schmerzen	<12>	Sicherheit im Umgang mit Informationen zur Sexualität und Gesundheit
<4>	Weniger Stress durch Sexualität	<13>	Erlebnisse von Sicherheit, Geborgenheit und Nähe
<5>	Wohlfühlen mit eigenem Körper und Sexualität	<14>	Sexualität als Möglichkeit zur Entspannung
<6>	Sexuelle Zufriedenheit	<15>	Teilnahme an sozialen Aktivitäten
<7>	Mehr Lust	<955 fixed>	Sonstiges
<8>	Mehr Zufriedenheit in der Partnerschaft	<966 fixed xor>	Keine
<9>	Mehr Kommunikation über Sexualität	<977 fixed xor>	Weiß nicht / keine Angabe

#PAGE 38

Base: Alle Befragten
*Question type: **Dyngrid***
#row order: randomize

[ch26b] Bitte beurteilen Sie jeden der folgenden vier Beziehungsstile unten in diesem Abschnitt nach dem Ausmaß, wie stark er für Sie zutrifft.

-[ch26b_1]	Mir fällt es leicht, anderen gefühlsmäßig nahe zu kommen. Ich fühle mich wohl dabei, wenn ich mich auf andere verlassen kann, und wenn sich andere auf mich verlassen. Ich mache mir keine Sorgen über das Alleinsein, oder darüber, dass andere mich nicht akzeptieren könnten.
-[ch26b_2]	Ich fühle mich wohl ohne enge gefühlsmäßige Beziehungen. Es ist sehr wichtig für mich, mich unabhängig und selbstständig zu fühlen, und ich bevorzuge es, nicht von anderen abhängig zu sein, oder dass andere von mir abhängig sind.
-[ch26b_3]	Ich möchte anderen gefühlsmäßig sehr nahe sein, aber ich bemerke oft, dass sich andere gegen so viel Nähe sträuben, wie ich sie mir wünschen würde. Ohne enge Beziehungen fühle ich mich unwohl, aber manchmal beunruhigt es mich, dass mich andere nicht so schätzen, wie ich sie.
-[ch26b_4]	Ich fühle mich unwohl dabei, anderen nahe zu kommen. Ich möchte gefühlsmäßig nahe Beziehungen, aber ich finde es schwierig, anderen vollständig zu vertrauen oder von ihnen abhängig zu sein. Ich fürchte mich davor, verletzt zu werden, wenn ich es mir erlaube, anderen zu nahe zu kommen.
<1>	Gar nicht zutreffend 1

<2>	2
<3>	3
<4>	Mittel<br>4
<5>	5
<6>	6
<7>	Sehr stark zutreffend<br>7

#PAGE 39

Base: Alle Befragten
Question type: ***Multiple***
#row order: randomize

[ch27] Welche der folgenden Punkte treffen auf Sie persönlich in den letzten 12 Monaten zu?
(Bitte wählen Sie alle zutreffenden Antwortmöglichkeiten aus.)

<1>	Ich trinke Alkohol max. 1x pro Woche	<11 if gender in ([2])>	Ich hatte eine Abtreibung
<2>	Ich bin Nichtraucher:in	<12>	Religion ist mir wichtig
<3>	Ich treibe Sport mind. 1x pro Woche	<13>	Mein Geschlecht entspricht nicht meinem bei Geburt zugewiesenen Geschlecht
<4>	Ich kann problemlos Treppen steigen bis in das 2. Stockwerk	<14>	Ich würde mich weder als Mann noch als Frau bezeichnen
<5>	Ich ernähre mich gesund	<15>	Es gab ein Ereignis, bei dem jemand gegen meinen Willen mit mir Sex gehabt hat (Geschlechtsverkehr oder oralen, analen Sex) oder es versucht hat
<6>	Ich nehme regelmäßig Medikamente wegen einer chronischen Krankheit	<16>	Ich leide unter Erkrankungen, die Auswirkungen auf mein Sexualleben haben
<7 if gender in ([2])>	Ich bin / war schwanger	<17>	Ich hatte Sex mit einer anderen Person
<8 if gender in ([2])>	Ich stille mein Kind	<18>	Ich habe mich selbst befriedigt / masturbiert
<9 if gender in ([2])>	Ich verhüte aktuell hormonell (z.B. Pille, Ring etc.)	<966 fixed xor>	Nichts davon
<10>	Mein Kind ist geboren	<977 fixed xor>	Weiß nicht / keine Angabe

#PAGE 40

Base: Alle Befragten
Question type: ***Multiple***
#row order: randomize

[ch28] Was trifft auf Sie und Ihre **zwischenmenschlichen Beziehungen** in den letzten 12 Monaten zu?
(Bitte wählen Sie alle zutreffenden Antwortmöglichkeiten aus.)

<1>	Mein:e Partner:in gibt mir Halt, Sicherheit und Geborgenheit	<11>	Ich hatte eine belastende Trennung (in den letzten 12 Monaten)
<2>	Mein:e Partner:in ist bei Stresssituationen eine große Unterstützung	<12>	Ich erlebe Konflikte in Beziehungen
<3>	Ich verbringe viel Zeit mit den Personen, die mir wichtig sind	<13>	Ich habe Gefühle von Einsamkeit
<4>	In Stresssituationen unterstützt mich mein:e Partner:in	<14>	Ich habe Gefühle von Wertlosigkeit
<5>	In Stresssituationen unterstützen mich meine engen Bezugspersonen	<15>	Ich erlebe Belastungen durch meinen Beruf
<6>	Ich mache meine Arbeit gerne	<16>	Mein:e Partner:in hat kein Interesse an meinen Gefühlen und Gedanken
<7>	Ich erledige den Großteil der Hausarbeit	<17>	Meine Freund:innen oder Familie haben kein Interesse an meinen Gefühlen und Gedanken
<8>	Ich erledige den Großteil der Fürsorgearbeit (Kinderbetreuung, Pflege)	<966 fixed xor>	Nichts davon
<9>	Ich kann belastende Erlebnisse gut verarbeiten	<977 fixed xor>	Weiß nicht / keine Angabe
<10>	Ich erlebe Diskriminierung wegen meiner Sexualität		

#PAGE 41

Base: Alle Befragten
Question type: ***Multiple***
#row order: randomize
#SPD Category: health

[ch29] Welche aktuellen und vergangenen **Erkrankungen** wurden bei Ihnen schon einmal von einem Arzt / einer Ärztin oder einem Psychologen / einer Psychologin diagnostiziert?
(Bitte wählen Sie alle zutreffenden Antwortmöglichkeiten aus.)

<1>	Brustkrebs	<16>	Lichen Sclerosus
<2 if gender in ([2])>	Gebärmutterhalskrebs	<17 if gender in ([2])>	Starke Schmerzen während der Periode
<29 if gender in ([1])>	Prostatakarzinom	<18 if gender in ([2])>	Starke psychische Beeinträchtigungen durch den Menstruationszyklus

<3 if gender in ([2])>	Gebärmutterkrebs	<19 if gender in ([2])>	Polyzystisches Ovarsyndrom (PCOS)
<4 if gender in ([2])>	Krebserkrankung der Vulva	<20>	Unfruchtbarkeit bzw. länger als 6 Monate unerfüllter Kinderwunsch
<5 if gender in ([2])>	Vulvodynie	<21 if gender in ([2])>	Chronische Unterleibsschmerzen
<6>	Vestibulodynie	<22>	Chronische Reizblase
<7 if gender in ([1])>	Störung der sexuellen Erregung	<23>	Inkontinenz
<30 if gender in ([1])>	Erektionsstörung	<24>	Beckenbodenschwäche
<8 if gender in ([2])>	Eierstockkrebs	<25>	Sexuell übertragbare Krankheiten (z.B. Chlamydien, Genitalwarzen)
<9>	Geschlechtsinkongruenz (keine Übereinstimmung mit dem biologischen oder bei Geburt zugewiesenem Geschlecht)	<26>	Depression
<10>	Intersexualität	<27>	Angststörung
<11>	Mangelndes sexuelles Interesse	<28>	Posttraumatische Belastungsstörung
<12>	Endometriose	<944 fixed>	Andere psychische Erkrankungen
<13>	Orgasmusstörung	<955 fixed>	Sonstige
<14 if gender in ([2])>	Schmerzstörung (Dyspareunie)	<966 fixed xor>	Keine
<31 if gender in ([1])>	Vorzeitiger Samenerguss	<977 fixed xor>	Weiß nicht / keine Angabe
<15 if gender in ([2])>	Vaginismus		

#PAGE 42

Base: Alle Befragten
*Question type: **Dyngrid***
#row order: randomize
#SPD Category: sex

[ch30b] Im Folgenden finden Sie eine Liste von Gefühlen und Problemen, die Menschen manchmal im Zusammenhang mit ihrer Sexualität erleben. Bitte lesen Sie jeden Punkt sorgfältig durch und wählen Sie die Antwort aus, die am besten beschreibt, wie oft Sie dieses Problem in den letzten 30 Tagen (einschließlich heute) gestört oder beunruhigt hat.

Wie häufig haben Sie sich / waren Sie ...

-[ch30b_1]	... belastet gefühlt durch Sex.	-[ch30b_9]	... bedauert wegen sexueller Probleme.
-[ch30b_2]	... unglücklich mit sexuellen Beziehungen.	-[ch30b_10]	... geschämt für sexuelle Probleme.
-[ch30b_3]	... schuldig gefühlt wegen sexueller Probleme.	-[ch30b_11]	... unzufrieden mit Ihrem Sexualleben.
-[ch30b_4]	... frustriert wegen sexueller Probleme.	-[ch30b_12]	... verärgert über Ihr Sexualleben.

- [ch30b_5] ... gestresst gefühlt wegen Sex.
- [ch30b_6] ... minderwertig gefühlt wegen sexueller Probleme.
- [ch30b_7] ... Sorgen über Sexualität gemacht.
- [ch30b_8] ... sexuell ungenügend gefühlt.
- [ch30b_13] ... gestört gefühlt wegen mangelnder Lust.
- [ch30b_14] ... beunruhigt gefühlt durch Probleme mit sexueller Erregung.
- [ch30b_15] ... frustriert gefühlt wegen des Orgasmus.
- [ch30b_16] ... beeinträchtigt gefühlt durch Schmerzen beim Sex.

<1>	Nie
<2>	Selten
<3>	Gelegentlich
<4>	Häufig
<5>	Immer

#PAGE 43

Base: Alle Befragten
Question type: ***Open***
#integer Only

[ch31] Angenommen, Sie hätten sexuelle Probleme und würden sich dadurch stark belastet fühlen.
Wie viel Geld wären Sie bereit, für eine wirksame Hilfe zu bezahlen?
(Bitte geben Sie eine ganze Zahl ein.)

Not Sure

#PAGE 44

Question type: ***PdI***
#Question display logic:
if pdl.marital_status.last > months(4) and updated

[marital_status] Geben Sie bitte Ihren derzeitigen Familienstand oder Beziehungsstatus an.

<1>	Verheiratet
<2>	Eingetragene Lebenspartnerschaft
<3>	Mit einem Partner/einer Partnerin zusammenlebend, jedoch nicht verheiratet oder in einer eingetragenen Lebenspartnerschaft
<4>	Alleinstehend
<5>	Geschieden
<6>	Verwitwet
<7>	Getrennt lebend, jedoch weiterhin gesetzlich verheiratet oder in einer eingetragenen Lebenspartnerschaft
<8>	In einer Beziehung, aber nicht zusammenlebend
<555 fixed>	Sonstiges
<666 fixed>	Ich möchte keine Angabe machen

#PAGE 45

*Question type: **Pdl***
#Question display logic:
if pdl.educ.last > months(12) and updated

[educ] Welchen höchsten Bildungsabschluss haben Sie?

<1>	Noch in schulischer Ausbildung
<2>	Sonder-/Förderschulabschluss
<3>	Haupt-(Volks-)schulabschluss
<4>	Abschluss der polytechnischen Oberschule
<5>	Realschul- oder gleichwertiger Abschluss
<6>	Fachhochschulreife
<7>	Abitur
<8>	Ohne Schulabschluss
<777>	Keine Angabe

#PAGE 46

*Question type: **Pdl***
#Question display logic:
if pdl.pinc.last>months(4) and updated

[pinc] Wie hoch ist Ihr *persönliches* Nettoeinkommen pro Monat? Hiermit ist Ihr persönliches Einkommen nach Abzug von Steuern und Sozialversicherungsabgaben gemeint.

<1>	unter EUR 500	<8>	EUR 3.500 bis unter EUR 4.000
<2>	EUR 500 bis unter EUR 1.000	<9>	EUR 4.000 bis unter EUR 4.500
<3>	EUR 1.000 bis unter EUR 1.500	<10>	EUR 4.500 bis unter EUR 5.000
<4>	EUR 1.500 bis unter EUR 2.000	<11>	EUR 5.000 bis unter EUR 10.000
<5>	EUR 2.000 bis unter EUR 2.500	<12>	EUR 10.000 und mehr
<6>	EUR 2.500 bis unter EUR 3.000	<13>	habe kein persönliches Einkommen
<7>	EUR 3.000 bis unter EUR 3.500	<777>	keine Angabe

#PAGE 47

*Question type: **Pdl***
#Question display logic:
if pdl.hinc.last>months(4) and updated

[hinc] Wie hoch ist das Nettoeinkommen *Ihres gesamten Haushaltes* pro Monat?

<1>	unter EUR 500	<8>	EUR 3.500 bis unter EUR 4.000
<2>	EUR 500 bis unter EUR 1.000	<9>	EUR 4.000 bis unter EUR 4.500
<3>	EUR 1.000 bis unter EUR 1.500	<10>	EUR 4.500 bis unter EUR 5.000

<4>	EUR 1.500 bis unter EUR 2.000	<11>	EUR 5.000 bis unter EUR 10.000
<5>	EUR 2.000 bis unter EUR 2.500	<12>	EUR 10.000 und mehr
<6>	EUR 2.500 bis unter EUR 3.000	<777>	keine Angabe
<7>	EUR 3.000 bis unter EUR 3.500		

#PAGE 48

*Question type: **PdI***
#Question display logic:
if pdl.emps.last>months(4) and updated

[emps] Welche der folgenden Beschreibungen trifft am besten auf Ihre derzeitige Erwerbssituation zu?

<1>	Erwerbstätigkeit/Berufstätigkeit
<2>	Arbeitslosengeld I
<3>	Renten, Pension
<4>	Angehörige
<5>	Vermögen, Vermietung, Zinsen
<6>	Laufende Hilfe zum Lebensunterhalt
<7>	Leistungen nach Hartz IV
<8>	Sonstige Unterstützungen (z.B. BaföG)
<9>	Elterngeld/Erziehungsgeld
<777>	keine Angabe

#PAGE 49

*Question type: **PdI***
#Question display logic:
if pdl.housz.last>months(4) and updated

[housz] Wie viele Personen, Sie eingeschlossen, leben in Ihrem Haushalt? (Kinder und Erwachsene insgesamt)

#PAGE 50

*Question type: **PdI***
#Question display logic:
if pdl.housz18.last>months(4) and updated

[housz18] Wie viele Kinder unter 18 Jahren leben in Ihrem Haushalt?

#PAGE 51

*Question type: **PdI***
#Question display logic:
if pdl.sexuality.last>months(12) and updated

[sexuality] Bitte geben Sie Ihre sexuelle Orientierung an. Sind Sie…?

<1>	heterosexuell
<2>	homosexuell (schwul/lesbisch)
<3>	bisexuell
<4>	sonstige
<777>	keine Angabe
<888>	weiß nicht

#PAGE 52

*Question type: **PdI***
#Question display logic:
if pdl.migration.last>months(12) and updated

[migration] Haben Sie einen Migrationshintergrund?
Unter Personen mit Migrationshintergrund versteht man alle nach 1949 auf das heutige Gebiet der Bundesrepublik Deutschland Zugewanderten, sowie alle in Deutschland geborenen Ausländer und alle in Deutschland als Deutsche Geborenen mit zumindest einem nach 1949 zugewanderten oder als Ausländer in Deutschland geborenen Elternteil.

<1>	ja
<2>	nein

#Question display logic:
if (not pdl.reli or (pdl.reli.last > months(12)))
#PAGE 53

*Question type: **PdI***
#Question display logic:
if (not pdl.reli or pdl.reli.last > months(12)) and updated

[reli] Welchem Glauben gehören Sie an?

<1>	röm.-katholisch
<2>	evangelisch-lutherisch
<3>	evangelisch-freikirchlich
<4>	orthodox-christlich
<5>	jüdisch
<6>	islamisch
<7>	sonstiger Glaube [open] please specify
<8>	keinem
<777>	keine Angabe

*Question type: **Text***

```
{
if q_relis: pdl.relis.set(q_relis)
}
```

#PAGE 54

*Question type: **PdI***
#Question display logic:
if pdl.urban.last>months(4) and updated

[urban] Würden Sie Ihr Wohnumfeld als städtisch, vorstädtisch oder ländlich bezeichnen?

<1>	Städtisch
<2>	Vorstädtisch
<3>	Ländlich
<777>	weiß nicht

#Question display logic:
if panman.is_panelist
#PAGE 55

###
####### redirect to PROFILES
###
{page profile2 if panman.is_panelist}
{goto survey profile_update_2019_DE resume=leer2}
{end page profile2}

#PAGE 56

*Question type: **Text***
#Question display logic:
if False

#PAGE 57

###
######################### QUALITY CHECK - mandatory for every survey run in the German panel
###

#Question display logic:
if panman.is_panelist
#PAGE 58

{page p_QC if panman.is_panelist}
{text}
<div class="question-text">
<p>Sie sind nun fast am Ende der Befragung angekommen. Wir sind bemüht, die Qualität

```
unserer Umfragen stetig zu verbessern und würden Sie bitten, kurz die Umfrage selbst zu bewerten.</p>
<p> </p>
</div>
{end text}
{module sample=1}
[qualityControl_understand] {scale 1 9 required=HARD} Wie leicht oder wie schwer waren die Fragen insgesamt zu verstehen?
 -[qualityControl_understand_scale] Die Fragen waren schwer zu verstehen|Die Fragen waren leicht zu verstehen
[qualityControl_interest] {scale 1 9 required=HARD} Wie interessant bzw. langweilig fanden Sie die Umfrage insgesamt?
 -[qualityControl_interest_scale] Ich fand die Umfrage langweilig|Ich fand die Umfrage interessant
{end module}
{module sample=1}
[qualityControl_fairness] {scale 1 9 required=HARD} Wie fair und neutral waren die Fragen insgesamt formuliert?
 -[qualityControl_fairness_scale] Die Fragen waren **nicht** fair und neutral|Die Fragen waren fair und neutral
[qualityControl_problems]{pdl-update qc03_problems required=HARD}
{end module}
[qualityControl_overall] {scale 1 9 required=HARD} Wie würden Sie den Fragebogen insgesamt bewerten?
 -[qualityControl_overall_scale] schlecht|ausgezeichnet
[comments] {open required=NONE rows=6 cols=60} Haben Sie weitere Anmerkungen oder Anregungen zu dieser Studie? Falls Sie eine Antwort wünschen, wenden Sie sich bitte direkt <a href="https://yougov.zendesk.com/hc/de/requests/new" target="_blank">hier</a>.
{
if qualityControl_understand_scale:
 pdl.qc_understand.set(qualityControl_understand_scale)
if qualityControl_interest_scale:
 pdl.qc_interest.set(qualityControl_interest_scale)
if qualityControl_fairness_scale:
 pdl.qc_fairness.set(qualityControl_fairness_scale)
if qualityControl_overall_scale:
 pdl.qc04_overall.set(qualityControl_overall_scale)
}
{end page p_QC}


#PAGE 59

################################################################################
#################### GRANTING POINTS to participants (QC-PDLs are set based on variables "team" and "points")
#################### in addtition, several variables are written, used for cleaning
################################################################################
```

#PAGE 60

{page punktevergabe}
{
if panman.is_panelist: panman.grant()
pdl.qc_team.set(team)
pdl.qc_points.set(points)
#https://datum.yougov.net/namespaces/panel:4/definitions/qc_team/
}
{end page punktevergabe}

#PAGE 61

*Question type: **Single***
#Question display logic:
if False

[Z_geschlecht] Zusatzfrage: Geschlecht

<1>	männlich
<2>	weiblich

*Question type: **Open***
#integer Only
#Question display logic:
if False

[Z_alter] Zusatzfrage: Alter

*Question type: **Single***
#Question display logic:
if False

[z_age_omnibus_16] Alter für den Omnibus ab 16

<1>	16 bis 24 Jahre
<2>	25 bis 34 Jahre
<3>	35 bis 44 Jahre
<4>	45 bis 54 Jahre
<5>	55 Jahre und älter

*Question type: **Single***
#Question display logic:
if False

[z_age_omnibus_18] Alter für den Omnibus ab 18

<1>	18 bis 24 Jahre
<2>	25 bis 34 Jahre
<3>	35 bis 44 Jahre
<4>	45 bis 54 Jahre
<5>	55 Jahre und älter

*Question type: **Single***
#Question display logic:
if False

[z_age_sex_omnibus_16] Kategorisierte Altersvariable mit Geschlecht gekreuzt (ab 16)

<1>	männlich 16-24 Jahre
<2>	weiblich 16-24 Jahre
<3>	männlich 25-34 Jahre
<4>	weiblich 25-34 Jahre
<5>	männlich 35-44 Jahre
<6>	weiblich 35-44 Jahre
<7>	männlich 45-54 Jahre
<8>	weiblich 45-54 Jahre
<9>	männlich 55 Jahre und älter
<10>	weiblich 55 Jahre und älter

*Question type: **Single***
#Question display logic:
if False

[z_age_sex_omnibus_18] Kategorisierte Altersvariable mit Geschlecht gekreuzt (ab 18)

<1>	männlich 18-24 Jahre
<2>	weiblich 18-24 Jahre
<3>	männlich 25-34 Jahre
<4>	weiblich 25-34 Jahre
<5>	männlich 35-44 Jahre
<6>	weiblich 35-44 Jahre
<7>	männlich 45-54 Jahre
<8>	weiblich 45-54 Jahre
<9>	männlich 55 Jahre und älter
<10>	weiblich 55 Jahre und älter

*Question type: **Text***

```
{
if pdl.gender: Z_geschlecht.set(pdl.gender)
if pdl.age:
 Z_alter.set(pdl.age)
 if (pdl.age > 15 and pdl.age < 25) or (pdl.age > 17 and pdl.age < 25):
  if pdl.age > 15 and pdl.age < 25 : z_age_omnibus_16.set(1)
  if pdl.age > 17 and pdl.age < 25 : z_age_omnibus_18.set(1)
 elif pdl.age > 24 and pdl.age < 35:
  z_age_omnibus_16.set(2)
  z_age_omnibus_18.set(2)
 elif pdl.age > 34 and pdl.age < 45:
  z_age_omnibus_16.set(3)
  z_age_omnibus_18.set(3)
 elif pdl.age > 44 and pdl.age < 55:
  z_age_omnibus_16.set(4)
  z_age_omnibus_18.set(4)
 elif pdl.age > 54:
  z_age_omnibus_16.set(5)
  z_age_omnibus_18.set(5)
if pdl.gender and pdl.age:
```

```
 if pdl.gender==1 and ((pdl.age > 15 and pdl.age < 25) or (pdl.age > 17 and pdl.age < 25)):
  if pdl.age > 15 and pdl.age < 25 : z_age_sex_omnibus_16.set(1)
  if pdl.age > 17 and pdl.age < 25 : z_age_sex_omnibus_18.set(1)
 elif pdl.gender==2 and ((pdl.age > 15 and pdl.age < 25) or (pdl.age > 17 and pdl.age < 25)):
  if pdl.age > 15 and pdl.age < 25 : z_age_sex_omnibus_16.set(2)
  if pdl.age > 17 and pdl.age < 25 : z_age_sex_omnibus_18.set(2)
 elif (pdl.age > 24 and pdl.age < 35) and pdl.gender==1:
  z_age_sex_omnibus_16.set(3)
  z_age_sex_omnibus_18.set(3)
 elif (pdl.age > 24 and pdl.age < 35) and pdl.gender==2:
  z_age_sex_omnibus_16.set(4)
  z_age_sex_omnibus_18.set(4)
 elif (pdl.age > 34 and pdl.age < 45) and pdl.gender==1:
  z_age_sex_omnibus_16.set(5)
  z_age_sex_omnibus_18.set(5)
 elif (pdl.age > 34 and pdl.age < 45) and pdl.gender==2:
  z_age_sex_omnibus_16.set(6)
  z_age_sex_omnibus_18.set(6)
 elif (pdl.age > 44 and pdl.age < 55) and pdl.gender==1:
  z_age_sex_omnibus_16.set(7)
  z_age_sex_omnibus_18.set(7)
 elif (pdl.age > 44 and pdl.age < 55) and pdl.gender==2:
  z_age_sex_omnibus_16.set(8)
  z_age_sex_omnibus_18.set(8)
 elif pdl.age > 54 and pdl.gender==1:
  z_age_sex_omnibus_16.set(9)
  z_age_sex_omnibus_18.set(9)
 elif pdl.age > 54 and pdl.gender==2:
  z_age_sex_omnibus_16.set(10)
  z_age_sex_omnibus_18.set(10)
}
```

Question type: ***Multiple***

[health_condition_self] {multiple columns = 2 varlabel="Diagnostizierte Erkrankungen" spd_category="health"}

Leiden Sie an einer oder mehreren der folgenden Beschwerden bzw. chronischen Krankheiten? (Mehrfachauswahl möglich)

<1> Alzheimer
<22> Andere Herz-Kreislauf-Erkrankungen
<21> Arterienverkalkung
<2> Arthrose
<5> Asthma oder COPD
<6> Autismus
<20> Bluthochdruck
<26> Chronische Schmerzen
<10> Demenz
<11> Diabetes
<13> Epilepsie
<27> Erektile Dysfunktion
<23> Fettstoffwechselstörungen, z.B. erhöhte Cholesterinwerte

<4> Gelenkentzündung
<3> Gelenkrheumathismus
<15> HIV/Aids
<28> Inkontinenz
<7> Krebs
<12> Legasthenie
<9> Mukoviszidose
<16> Multiple Sklerose
<17> Osteoporose
<18> Parkinson
<29> Vorzeitige Ejakulation
<32> Saures Aufstoßen
<30> Schlaganfall
<31> Urinale Probleme
<24> Psoriasis (Schuppenflechte)
<19> Psychische Erkrankung(en)
<25> Rheumatische Leiden
<14> Schwerhörigkeit
<8> Zerebrale Lähmung
<555> Sonstige
<666 xor> Keine der genannten
<888 xor> weiß nicht
<777 xor> möchte ich nicht angeben
