# Supplementary material for: Assessment of Mental and Chronic Health Conditions as Determinants of Health Care Needs and Digital Innovations for Women With Sexual Dysfunction: Cross-Sectional Population-Based Survey Study in Germany
Source: J Particip Med. 2025 Aug 27;17:e71301. doi: 10.2196/71301 (PMC12386550; doi:10.2196/71301)
Supplement: Multimedia Appendix 2 [file jopm-v17-e71301-s002.pdf]

# Multimedia Appendix 2

**Corresponding Author:**
Laura Hatzler
Institute of Sexology and Sexual Medicine
Charité-Universitätsmedizin Berlin
Charité Platz 1, 10117 Berlin, Germany
Phone: +49 30 450 617 139
Fax: +49 30 529 992
E-Mail: laura.hatzler@charite.de

**Table S1.** Characteristics of the study population by comorbid CHC subgroups.
**Table S2.** Multivariable logistic regression model for SD with interaction term CHC*relationship (Model 1), in single women only (Model 2), and in partnered women only (Model 3).
**Table S3.** Comparison of the age and gender characteristics of the unweighted and weighted YouGov samples with the 2014 German Microcensus.
**Table S4.** Comparison of federal state characteristics of the unweighted and weighted YouGov samples with the 2014 German Microcensus.
**Table S5.** Multivariable logistic regression models for FSDS-DAO scores using the binary CHC status (Model 1), MH status (Model 2), and comorbid CHC subgroups (Model 3).
**Table S6.** Weighted adjacency matrix for network A with CHC subgroups and total SP.
**Table S7.** Weighted adjacency matrix for network B with CHC subgroups and total SD.
**Table S8.** Weighted adjacency matrix for network C with CHC subgroups and individual domains of SP.
**Table S9.** Weighted adjacency matrix for network D with CHC subgroups and individual domains of SD.
**Table S10.** Threshold parameters for different network models.
**Table S11**. Help-seeking behavior in women with SD among CHC status, MH status, and comorbid CHC subgroups.
**Table S12**. Healthcare preferences and needs of women with SD among CHC status, MH status, and comorbid CHC subgroups.

**Table S1.** Characteristics of the study population by comorbid CHC subgroups.[a]

| | MH+ (n=528) | CV+ (n=528) | GY+ (n=506) | IN+ (n=241) | CA+ (n=118) | PA+ (n=278) | NE+ (n=79) |
|---|---|---|---|---|---|---|---|
| **Sociodemographics, n (%)** | | | | | | | |
| Age, mean (SD), y | 49.0 (14.3) | 59.1 (12.2) | 49.9 (15.1) | 56.0 (12.2) | 56.1 (13.6) | 54.3 (13.5) | 51.1 (14.6) |
| Age groups, y | | | | | | | |
| 18-30 | 77 (14.6) | 25 (4.8) | 77 (15.3) | 20 (8.4) | 11 (9.2) | 18 (6.6) | 11 (14.5) |
| 31-40 | 67 (12.6) | 48 (9.1) | 93 (18.4) | 42 (17.4) | 18 (15.2) | 44 (15.7) | 9 (11.9) |
| 41-50 | 104 (19.7) | 264 (50.0) | 186 (36.8) | 113 (46.9) | 51 (43.1) | 144 (51.8) | 39 (49.3) |
| 51-65 | 228 (43.1) | 170 (32.2) | 83 (16.3) | 48 (20.1) | 32 (27.0) | 58 (20.8) | 11 (13.3) |
| >65 | 53 (10.1) | 21 (4.0) | 67 (13.2) | 17 (7.2) | 7 (5.5) | 14 (5.1) | 9 (11.0) |
| Education, ≥12 years | 227 (43.0) | 161 (30.5) | 219 (43.3) | 94 (39.1) | 46 (39.2) | 95 (34.3) | 29 (36.6) |
| Monthly netto income, EUR | | | | | | | |
| <€2500 | 402 (85.4) | 397 (85.2) | 363 (80.5) | 179 (82.8) | 80 (78.1) | 207 (83.9) | 57 (82.9) |
| €2500-5000 | 66 (13.9) | 59 (12.6) | 82 (18.1) | 35 (16.3) | 22 (21.9) | 38 (15.3) | 12 (17.1) |
| >€5000 | 3 (0.6) | 10 (2.2) | 6 (1.3) | 2 (0.9) | 0 (0) | 2 (0.8) | 0 (0) |
| Employed | 219 (44.3) | 162 (31.9) | 225 (46.4) | 93 (40.2) | 37 (34.2) | 82 (30.3) | 21 (26.4) |
| Religious | 278 (54.4) | 318 (62.8) | 298 (61.2) | 144 (62.0) | 72 (63.5) | 160 (59.6) | 47 (60.9) |
| Relationship | 295 (56.1) | 298 (56.7) | 311 (61.6) | 145 (60.2) | 67 (56.5) | 154 (55.4) | 43 (55.0) |
| Heterosexual | 449 (89.3) | 454 (92.4) | 435 (90.9) | 207 (91.7) | 100 (90.6) | 238 (92.9) | 59 (87.1) |
| Migration background | 77 (14.6) | 57 (10.9) | 80 (15.9) | 29 (11.9) | 12 (10.5) | 29 (10.3) | 14 (18.0) |
| Children, ≥1 in same household | 96 (18.3) | 62 (11.8) | 119 (23.6) | 39 (16.2) | 20 (16.9) | 41 (14.8) | 14 (17.6) |
| Delivery[b] | 24 (4.7) | 25 (4.9) | 39 (7.7) | 14 (5.9) | 5 (4.4) | 15 (5.5) | 2 (2.8) |
| Breastfeeding[b] | 8 (1.6) | 6 (1.1) | 8 (1.6) | 2 (0.8) | 1 (1.0) | 1 (0.3) | 1 (1.1) |
| Household size, ≥2 | 341 (64.6) | 334 (63.2) | 362 (71.6) | 170 (70.7) | 70 (59.5) | 165 (59.2) | 55 (70.1) |
| Majority of housework | 285 (55.8) | 279 (55.9) | 298 (60.1) | 151 (66.7) | 62 (55.0) | 149 (56.5) | 29 (40.1) |
| Primary caregiver | 63 (12.4) | 38 (7.7) | 91 (18.3) | 31 (13.5) | 11 (9.9) | 30 (11.5) | 11 (14.9) |
| Urban area | 229 (43.3) | 202 (38.3) | 193 (38.2) | 94 (39.2) | 42 (35.2) | 105 (37.8) | 29 (37.1) |
| **Behavioral risk factor, n (%)** | | | | | | | |
| Medication for chronic condition | 269 (52.0) | 310 (61.0) | 230 (46.0) | 177 (65.2) | 63 (55.9) | 131 (56.3) | 39 (52.7) |
| Alcohol consumption[c] | 394 (76.1) | 110 (21.7) | 125 (24.9) | 56 (24.0) | 28 (24.5) | 59 (21.5) | 9 (12.4) |
| Smoking | 262 (50.6) | 243 (47.8) | 225 (44.9) | 114 (49.1) | 55 (48.3) | 136 (50.2) | 43 (58.1) |

| | | | | | | | |
|---|---|---|---|---|---|---|---|
| Low physical activity[c] | 347 (65.6) | 376 (71.3) | 327 (64.6) | 147 (61.0) | 78 (65.8) | 186 (66.9) | 58 (73.3) |
| Sexual discrimination | 7 (1.4) | 4 (0.8) | 10 (2.0) | 1 (0.4) | 1 (0.7) | 3 (1.0) | 2 (2.7) |
| **Sexual Behavior, n (%)** | | | | | | | |
| Masturbation[b] | 231 (44.7) | 156 (30.7) | 218 (43.5) | 91 (39.3) | 36 (31.5) | 88 (32.3) | 21 (28.8) |
| Partnered sexual activity[b] | 120 (23.3) | 55 (10.8) | 111 (22.2) | 39 (16.8) | 13 (11.5) | 38 (14.2) | 9 (11.6) |
| Sexual trauma[b] | 54 (10.4) | 30 (5.8) | 44 (8.7) | 17 (7.1) | 7 (6.1) | 21 (7.6) | 5 (6.4) |
| Spending time in close relationships[b] | 193 (37.7) | 213 (42.6) | 227 (45.8) | 101 (44.8) | 42 (37.2) | 110 (41.7) | 28 (38.7) |

Abbreviation: CHC, chronic health conditions. MH+, comorbid mental CHC. CV+, comorbid cardiovascular and metabolic CHC. GY+, comorbid gynecological CHC. IN+, comorbid infectious and inflammatory CHC. CA+, comorbid cancer CHC. PA+, comorbid pain-related CHC. NE+, comorbid neurological CHC.

[a]weighted frequencies, group sizes are unweighted.

[b]in the past 12 months.

c<once per week.

**Table S2.** Multivariable logistic regression model for SD with interaction term CHC*relationship.

| | **Model (n=1675)** | |
|---|---|---|
| | OR (95% CI) | *P* value |
| CHC [yes] | 4.18 (2.40-7.68) | <.001 |
| Age | 0.96 (0.95-0.97) | <.001 |
| Sexual activity[a] | 0.90 (0.64-1.25) | .555 |
| Relationship | 2.27 (1.29-4.21) | .006 |
| CHC*Relationship | 0.49 (0.25-0.95) | .039 |

Abbreviation: SD, sexual dysfunction, CHC, chronic health conditions.
[a]Partnered sexual activity in the last 12 months.

**Table S3.** Comparison of the age and gender characteristics of the unweighted and weighted YouGov samples with the 2014 German Microcensus.[a]

| | **Microcensus 2014** | | | **YouGov data** | | | | | |
|---|---|---|---|---|---|---|---|---|---|
| **Age and gender, %** | | | | **Weighted sample** | | | **Unweighted sample** | | |
| | male | female | total | male | female | total | male | female | total |
| 18-24 | 4.7 | 4.4 | 9.1 | 4.7 | 4.4 | 9.1 | 3.9 | 4.7 | 8.6 |
| 25-34 | 7.7 | 7.4 | 15.0 | 7.7 | 7.4 | 15.0 | 7.3 | 7.8 | 15.1 |
| 35-44 | 7.4 | 7.2 | 14.6 | 7.4 | 7.2 | 14.6 | 7.6 | 7.6 | 15.2 |
| 45-54 | 10.1 | 9.9 | 19.9 | 10.1 | 9.9 | 19.9 | 9.0 | 9.9 | 18.9 |
| 55-90 | 18.8 | 22.5 | 41.3 | 18.8 | 22.5 | 41.3 | 20.1 | 22.2 | 42.3 |
| Total | 48.6 | 51.4 | 100.0 | 48.6 | 51.4 | 100.0 | 47.8 | 52.2 | 100.0 |

[a] Federal Statistical Office, editors. Microcensus 2014. Wiesbaden, Germany; 2014. doi:10.21242/12211.2014.00.00.1.2.1

**Table S4.** Comparison of federal state characteristics of the unweighted and weighted YouGov samples with the 2014 German Microcensus.[a]

| | **Microcensus 2014** | **YouGov data** | |
|---|---|---|---|
| **Federal states, %** | | **Weighted sample** | **Unweighted sample** |
| Baden-Wuerttemberg | 13.1 | 13.1 | 12.2 |
| Bavaria | 15.6 | 15.6 | 14.6 |
| Berlin | 4.3 | 4.3 | 4.4 |
| Brandenburg | 3.1 | 3.1 | 3.0 |
| Bremen | 0.8 | 0.8 | 1.0 |
| Hamburg | 2.2 | 2.2 | 2.6 |
| Hesse | 7.5 | 7.5 | 7.7 |
| Mecklenburg-Western Pomerania | 2.0 | 2.0 | 2.5 |
| Lower Saxony | 9.6 | 9.6 | 8.7 |
| North Rhine-Westphalia | 21.6 | 21.6 | 22.3 |
| Rhineland-Palatinate | 4.9 | 4.9 | 4.8 |
| Saarland | 1.2 | 1.2 | 1.5 |
| Saxony | 5.1 | 5.1 | 5.6 |
| Saxony-Anhalt | 2.8 | 2.8 | 2.6 |
| Schleswig-Holstein | 3.5 | 3.5 | 4.1 |
| Thuringia | 2.7 | 2.7 | 2.4 |

[a] Federal Statistical Office, editors. Microcensus 2014. Wiesbaden, Germany; 2014. doi:10.21242/12211.2014.00.00.1.2.1

**Table S5.** Multivariable linear regression models for FSDS-DAO scores using the binary CHC status (Model 1), MH status (Model 2), and comorbid CHC subgroups (Model 3).

| | **Model 1 (n=1700)** | | **Model 2 (n=1049)** | | **Model 3 (n=1515)** | |
|---|---|---|---|---|---|---|
| | Estimate (95% CI) | *P* value | Estimate (95% CI) | *P* value | Estimate (95% CI) | *P* value |
| (Intercept) | 17.9 (15.7 – 20.0) | <0.001 | 21.4 (18.3 – 24.6) | <0.001 | 18.1 (15.7 – 20.4) | <0.001 |
| CHC [yes] | 6.0 (4.8-7.2) | <.001 | | | | |
| Age | -0.2 (-0.3 – -0.2) | <.001 | -0.2 (-0.3 – -0.2) | <0.001 | -0.2 (-0.3 – -0.2) | <.001 |
| Sexual activity[a] | -1.1 (-2.6 – 0.4) | .146 | -2.6 (-4.6 – -0.6) | .010 | -1.8 (-3.4 – -0.3) | .022 |
| Relationship | 1.5 (0.4 – 2.7) | .010 | 1.7 (0.2 – 3.2) | .029 | 2.0 (0.8 – 3.2) | .001 |
| **MH+/CHC MH- [MH]** | | | 5.0 (3.5 – 6.5) | <.001 | | |
| **CHC** | | | | | | |
| MH+ | | | | | 5.8 (4.5 – 7.1) | <.001 |
| CV+ | | | | | 2.4 (1.0 – 3.8) | .001 |
| GY+ | | | | | 2.6 (1.3 – 3.9) | <.001 |
| IN+ | | | | | 0.4 (-1.3 – 2.1) | .645 |
| CA+ | | | | | 2.9 (0.6 – 5.2) | .014 |
| PA+ | | | | | 0.9 (-0.8 – 2.6) | .325 |
| NE+ | | | | | -1.6 (-4.6 – 1.3) | .279 |
| $R^2$ | .111 | | .113 | | .152 | |

Abbreviation: FSDS-DAO, Female Sexual Distress Scale-Desire/Arousal/Orgasm, CHC, chronic health conditions. MH, mental CHC. CV, cardiovascular and metabolic CHC. GY, gynecological CHC. IN, infectious and inflammatory CHC. CA, Cancer CHC. PA, pain-related CHC. NE, neurological CHC. PH, physical CHC.

[a]Partnered sexual activity in the last 12 months.

**Table S6.** Weighted adjacency matrix for network A with CHC subgroups and total SP.

| | **SP** | **CA** | **CV** | **NE** | **PA** | **IN** | **MH** | **GY** |
|---|---|---|---|---|---|---|---|---|
| **SP** | - | 0.0 | 0.0 | 0.0 | 0.0 | 0.0 | 0.42 | 0.59 |
| **CA** | 0.0 | - | 0.52 | 0.0 | 0.43 | 0.0 | 0.0 | 0.0 |
| **CV** | 0.0 | 0.52 | - | 0.19 | 0.99 | 0.46 | 0.16 | 0.14 |
| **NE** | 0.0 | 0.0 | 0.19 | - | 0.31 | 0.0 | 0.0 | 0.0 |
| **PA** | 0.0 | 0.43 | 0.99 | 0.31 | - | 1.01 | 0.88 | 0.62 |
| **IN** | 0.0 | 0.0 | 0.46 | 0.0 | 1.01 | - | 0.18 | 0.39 |
| **MH** | 0.42 | 0.0 | 0.16 | 0.0 | 0.88 | 0.18 | - | 0.77 |
| **GY** | 0.59 | 0.0 | 0.14 | 0.0 | 0.62 | 0.39 | 0.77 | - |

Abbreviation: SP, Sexual Problems. MH, Mental CHC. CV, Cardiovascular and metabolic CHC. GY, gynecological CHC. PA, pain. IN, infectious and inflammatory CHC. CA, Cancer CHC. NE, neurological CHC.

**Table S7.** Weighted adjacency matrix for network B with CHC subgroups and total SD.

| | **SD** | **CA** | **CV** | **NE** | **PA** | **IN** | **MH** | **GY** |
|---|---|---|---|---|---|---|---|---|
| **SD** | - | 0.32 | 0.0 | 0.0 | 0.0 | 0.0 | 0.75 | 0.32 |
| **CA** | 0.32 | - | 0.46 | 0.0 | 0.42 | 0.0 | 0.0 | 0.0 |
| **CV** | 0.0 | 0.46 | - | 0.0 | 0.9 | 0.35 | 0.09 | 0.08 |
| **NE** | 0.0 | 0.0 | 0.0 | - | 0.24 | 0.0 | 0.0 | 0.0 |
| **PA** | 0.0 | 0.42 | 0.9 | 0.24 | - | 1.04 | 0.77 | 0.55 |
| **IN** | 0.0 | 0.0 | 0.35 | 0.0 | 1.04 | - | 0.21 | 0.4 |
| **MH** | 0.75 | 0.0 | 0.09 | 0.0 | 0.77 | 0.21 | - | 0.79 |
| **GY** | 0.32 | 0.0 | 0.08 | 0.0 | 0.55 | 0.4 | 0.79 | - |

Abbreviation: SD, Sexual Dysfunction. MH, Mental CHC. CV, Cardiovascular and metabolic CHC. GY, gynecological CHC. PA, pain. IN, infectious and inflammatory CHC. CA, Cancer CHC. NE, neurological CHC.

**Table S8.** Weighted adjacency matrix for network C with CHC subgroups and individual domains of SP.

| | SP1 | SP2 | SP3 | SP4 | CA | CV | NE | PA | IN | MH | GY |
|---|---|---|---|---|---|---|---|---|---|---|---|
| **SP1** | - | 2.45 | 0.66 | 0.53 | 0.0 | 0.0 | 0.0 | 0.0 | 0.0 | 0.13 | 0.35 |
| **SP2** | 2.45 | - | 2.13 | 0.95 | 0.15 | 0.0 | 0.0 | 0.0 | 0.0 | 0.0 | 0.0 |
| **SP3** | 0.66 | 2.13 | - | 0.95 | 0.0 | 0.0 | 0.0 | 0.0 | 0.0 | 0.05 | 0.03 |
| **SP4** | 0.53 | 0.95 | 0.95 | - | 0.0 | -0.23 | 0.0 | 0.0 | 0.0 | 0.0 | 0.21 |
| **CA** | 0.0 | 0.15 | 0.0 | 0.0 | - | 0.56 | 0.0 | 0.29 | 0.0 | 0.0 | 0.0 |
| **CV** | 0.0 | 0.0 | 0.0 | -0.23 | 0.56 | - | 0.21 | 0.9 | 0.36 | 0.08 | 0.17 |
| **NE** | 0.0 | 0.0 | 0.0 | 0.0 | 0.0 | 0.21 | - | 0.21 | 0.0 | 0.0 | 0.0 |
| **PA** | 0.0 | 0.0 | 0.0 | 0.0 | 0.29 | 0.9 | 0.21 | - | 0.97 | 0.69 | 0.51 |
| **IN** | 0.0 | 0.0 | 0.0 | 0.0 | 0.0 | 0.36 | 0.0 | 0.97 | - | 0.0 | 0.35 |
| **MH** | 0.13 | 0.0 | 0.05 | 0.0 | 0.0 | 0.08 | 0.0 | 0.69 | 0.0 | - | 0.69 |
| **GY** | 0.35 | 0.0 | 0.03 | 0.21 | 0.0 | 0.17 | 0.0 | 0.51 | 0.35 | 0.69 | - |

Abbreviation: SP, Sexual Problems. SP1, Sexual Desire Problems. SP2, Sexual Arousal Problems. SP3, Orgasm Problems. SP4, Sexual Pain Problems. MH, Mental CHC. CV, Cardiovascular and metabolic CHC. GY, gynecological CHC. PA, pain. IN, infectious and inflammatory CHC. CA, Cancer CHC. NE, neurological CHC.

**Table S9.** Weighted adjacency matrix for network D with CHC subgroups and individual domains of SD.

| | SD1 | SD2 | SD3 | SD4 | CA | CV | NE | PA | IN | MH | GY |
|---|---|---|---|---|---|---|---|---|---|---|---|
| **SD1** | - | 2.91 | 1.03 | 0.83 | 0.0 | 0.0 | 0.0 | 0.0 | 0.0 | 0.15 | 0.0 |
| **SD2** | 2.91 | - | 2.79 | 0.88 | 0.0 | 0.0 | 0.0 | 0.0 | 0.0 | 0.52 | 0.0 |
| **SD3** | 1.03 | 2.79 | - | 1.79 | 0.0 | 0.0 | 0.0 | 0.0 | 0.0 | 0.0 | 0.11 |
| **SD4** | 0.83 | 0.88 | 1.79 | - | 0.0 | 0.0 | 0.0 | 0.0 | 0.0 | 0.18 | 0.0 |
| **CA** | 0.0 | 0.0 | 0.0 | 0.0 | - | 0.12 | 0.0 | 0.2 | 0.0 | 0.0 | 0.0 |
| **CV** | 0.0 | 0.0 | 0.0 | 0.0 | 0.12 | - | 0.87 | 0.24 | 0.09 | 0.09 | 0.04 |
| **NE** | 0.0 | 0.0 | 0.0 | 0.0 | 0.0 | 0.87 | - | 0.25 | 0.0 | 0.0 | 0.0 |
| **PA** | 0.0 | 0.0 | 0.0 | 0.0 | 0.2 | 0.24 | 0.25 | - | 0.99 | 0.8 | 0.48 |
| **IN** | 0.0 | 0.0 | 0.0 | 0.0 | 0.0 | 0.09 | 0.0 | 0.99 | - | 0.09 | 0.32 |
| **MH** | 0.15 | 0.52 | 0.0 | 0.18 | 0.0 | 0.0 | 0.0 | 0.8 | 0.09 | - | 0.79 |
| **GY** | 0.0 | 0.0 | 0.11 | 0.0 | 0.0 | 0.04 | 0.0 | 0.48 | 0.32 | 0.79 | - |

Abbreviation: SD, Sexual Dysfunction. SD1, Hypoactive Sexual Desire Disorder. SD2, Sexual Arousal Disorder. SD3, Orgasmic Disorder. SD4, Sexual Pain and Penetration Disorder. MH, Mental CHC. CV, Cardiovascular and metabolic CHC. GY, gynecological CHC. PA, pain. IN, infectious and inflammatory CHC. CA, Cancer CHC. NE, neurological CHC.

**Table S10.** Threshold parameters for network models (A-D).

| | Threshold | | | |
|---|---|---|---|---|
| **Symptom** | **Network A** | **Network B** | **Network C** | **Network D** |
| SP | 0.60 | | | |
| SP1 | | | -1.12 | |
| SP2 | | | -3.44 | |
| SP3 | | | -1.58 | |
| SP4 | | | -2.14 | |
| SD | | -2.04 | | |
| SD1 | | | | -3.87 |
| SD2 | | | | -4.40 |
| SD3 | | | | -3.12 |
| SD4 | | | | -3.07 |
| MH | -1.64 | -1.44 | -1.24 | -1.41 |
| CV | -1.20 | -1.04 | -1.14 | -1.01 |
| GY | -1.79 | -1.40 | -1.73 | -1.32 |
| IN | -2.36 | -2.37 | -2.17 | -2.16 |
| CA | -2.84 | -3.00 | -2.85 | -2.64 |
| PA | -2.95 | -2.78 | -2.71 | -2.77 |
| NE | -3.15 | -3.12 | -3.11 | -3.11 |

Abbreviation: SP, Sexual Problems. SP1, Sexual Desire Problems. SP2, Sexual Arousal Problems. SP3, Orgasm Problems. SP4, Sexual Pain Problems. SD, Sexual Dysfunction. SD1, Hypoactive Sexual Desire Disorder. SD2, Sexual Arousal Disorder. SD3, Orgasmic Disorder. SD4, Sexual Pain and Penetration Disorder. MH, Mental CHC. CV, Cardiovascular and metabolic CHC. GY, gynecological CHC. PA, pain. IN, infectious and inflammatory CHC. CA, Cancer CHC. NE, neurological CHC.

**Table S11**. Help-seeking behavior in women with SD among CHC status, MH status, and comorbid CHC subgroups.

| | no CHC (n=69) | CHC (n=207) | OR[a] | MH+ (n=127) | CHC MH- (n=78) | CV+ (n=75) | GY+ (n=104) | IN+ (n=43) | CA+ (n=26) | PA+ (n=51) | NE + (n=12) |
|---|---|---|---|---|---|---|---|---|---|---|---|
| **Treatment, n (%)** | 39 | 145 | | 96 | 49 | 56 | 72 | 28 | 14 | 37 | 9 |
| Received Treatment | 3 (7.0) | 16 (11.0) | 1.66 (0.50-7.92) | 13 (13.9) | 3 (5.5) | 8 (13.7) | 6 (8.9) | 5 (16.7) | 2 (13.1) | 5 (14.4) | 1 (11.2) |
| **Time to access n (%)** | 3 | 16 | | 13 | 3 | 8 | 6 | 5 | 2 | 5 | 1 |
| Less than 1 month | 0 (0) | 2 (11.7) | | 2 (14.1) | 0 (0) | 2 (24.6) | 0 (0) | 1 (18.4) | 1 (54.3) | 0 (0) | 0 (0) |
| 1-2 months | 0 (0) | 4 (22.9) | | 2 (14.8) | 2 (63.4) | 2 (24.4) | 3 (42.6) | 0 (0) | 1 (45.7) | 1 (15.5) | 1 (100.0) |
| 3-4 months | 0 (0) | 4 (23.2) | | 3 (20.5) | 1 (36.6) | 1 (12.8) | 2 (26.6) | 0 (0) | 0 (0) | 1 (13.4) | 0 (0) |
| 5-6 months | 2 (67.5) | 2 (12.6) | | 2 (15.2) | 0 (0) | 1 (13.5) | 1 (15.6) | 1 (21.2) | 0 (0) | 2 (37.6) | 0 (0) |
| Longer than 6 months | 1 (32.5) | 5 (29.6) | | 5 (35.5) | 0 (0) | 2 (24.6) | 1 (15.3) | 3 (60.3) | 0 (0) | 2 (33.5) | 0 (0) |
| **Information sources, n (%)** | 39 | 150 | | 102 | 48 | 58 | 76 | 29 | 14 | 38 | 8 |
| Internet | 23 (59.7) | 63 (42.1) | 0.49 (0.24-1.00) | 45 (44.8) | 18 (37.2) | 21 (36.4) | 40 (52.7) | 13 (46.1) | 5 (37.9) | 16 (42.5) | 2 (21.5) |
| Literature | 1 (2.7) | 22 (14.8) | 6.31 (1.30-102.58) | 14 (14.2) | 8 (16.3) | 8 (14.1) | 13 (17.4) | 4 (13.0) | 2 (13.5) | 1 (2.6) | 0 (0) |
| Partner(s) | 10 (25.5) | 42 (28.2) | 1.14 (0.53-2.64) | 28 (27.8) | 14 (29.3) | 16 (27.2) | 24 (30.9) | 8 (26.6) | 2 (17.0) | 9 (24.4) | 3 (33.4) |
| Friend(s) | 5 (12.9) | 40 (26.7) | 2.46 (0.98-7.45) | 32 (31.2) | 8 (17.6) | 18 (30.8) | 26 (33.4) | 9 (32.7) | 3 (19.0) | 10 (25.0) | 1 (12.2) |
| Support Groups | 6 (15.3) | 12 (8.1) | 0.49 (0.18-1.47) | 7 (7.3) | 5 (9.8) | 5 (8.2) | 6 (8.3) | 0 (0) | 0 (0) | 3 (6.8) | 0 (0) |
| General practitioner | 1 (2.1) | 12 (8.1) | 4.11 (0.70-114.44) | 8 (8.0) | 4 (8.4) | 7 (12.1) | 4 (5.0) | 5 (17.2) | 0 (0) | 4 (10.5) | 0 (0) |
| Gynecologists | 15 (38.0) | 61 (40.5) | 1.11 (0.54-2.31) | 39 (38.2) | 21 (44.2) | 25 (43.4) | 33 (43.1) | 11 (39.3) | 8 (53.6) | 20 (53.1) | 3 (35.7) |
| Urologist | 0 (0) | 7 (4.6) | NA[b] | 4 (4.0) | 3 (6.2) | 3 (5.1) | 4 (5.1) | 1 (3.4) | 1 (6.6) | 6 (15.6) | 0 (0) |
| **Dialog partners, n (%)** | 151 | 39 | | 101 | 50 | 58 | 76 | 29 | 14 | 39 | 9 |
| Partner(s) | 18 (46.9) | 69 (45.4) | 0.94 (0.47-1.91) | 46 (45.9) | 22 (45.2) | 25 (43.0) | 39 (51.2) | 13 (43.3) | 3 (23.3) | 17 (43.6) | 5 (54.3) |
| Family | 0 (0) | 14 (9.0) | NA[b] | 10 (9.7) | 4 (7.8) | 5 (9.1) | 9 (11.1) | 4 (13.6) | 0 (0) | 3 (7.6) | 2 (21.6) |
| Friend(s) | 9 (22.8) | 37 (24.5) | 1.10 (0.49-2.63) | 29 (28.6) | 7 (14.9) | 11 (19.7) | 22 (28.7) | 6 (21.3) | 3 (19.0) | 9 (23.6) | 1 (10.9) |
| Peer network | 0 (0) | 0 (0) | NA | 0 (0) | 0 (0) | 0 (0) | 0 (0) | 0 (0) | 0 (0) | 0 (0) | 0 (0) |
| General practitioner | 1 (2.6) | 11 (7.4) | 2.98 (0.57-51.62) | 9 (9.3) | 2 (3.7) | 5 (8.5) | 5 (6.4) | 4 (14.3) | 1 (7.1) | 4 (10.2) | 0 |
| Gynecologists | 11 (27.6) | 58 (38.2) | 1.62 (0.77-3.62) | 34 (33.4) | 24 (48.5) | 23 (40.0) | 31 (40.3) | 11 (36.5) | 7 (47.0) | 19 (47.3) | 2 (21.1) |
| Urologist | 0 (0) | 7 (4.4) | NA[b] | 5 (4.7) | 2 (3.9) | 2 (3.3) | 4 (4.7) | 2 (6.1) | 0 (0) | 3 (7.8) | 0 (0) |
| Psychiatrist | 1 (2.3) | 7 (4.9) | 2.19 (0.36-50.63) | 7 (6.5) | 1 (1.7) | 4 (6.3) | 4 (5.6) | 3 (8.9) | 0 (0) | 2 (5.1) | 0 (0) |
| Ohter physician | 1 (2.3) | 4 (2.7) | 1.21 (0.17-28.99) | 3 (2.9) | 1 (2.4) | 2 (3.7) | 2 (2.3) | 1 (3.0) | 0 (0) | 3 (8.2) | 0 (0) |
| Psychotherapist | 0 (0) | 22 (14.4) | NA[b] | 19 (18.8) | 3 (5.8) | 7 (11.9) | 16 (20.6) | 8 (27.0) | 0 (0) | 7 (17.6) | 0 (0) |
| **Received offerings, n (%)** | 38 | 149 | | 101 | 48 | 57 | 76 | 29 | 38 | 14 | 8 |
| Gynecological exam | 5 (12.9) | 33 (22.1) | 1.92 (0.75-5.94) | 18 (18.0) | 14 (29.3) | 21 (36.5) | 18 (23.2) | 6 (19.8) | 2 (12.9) | 10 (27.2) | 1 (12.2) |
| Urologist exam | 0 (0) | 0 (0) | NA[b] | 0 (0) | 0 (0) | 0 (0) | 0 (0) | 0 (0) | 0 (0) | 0 (0) | 0 (0) |
| Other physician exams | 1 (2.3) | 4 (2.7) | 1.18 (0.16-29.19) | 2 (2.1) | 2 (4.1) | 1 (1.9) | 4 (5.3) | 1 (3.1) | 0 (0) | 1 (2.8) | 0 (0) |
| Systemic hormone therapy | 3 (7.1) | 19 (12.6) | 1.87 (0.58-8.84) | 12 (12.2) | 7 (13.6) | 8 (13.4) | 12 (15.9) | 6 (19.5) | 0 (0) | 4 (9.7) | 1 (12.5) |
| Local hormone therapy | 0 (0) | 12 (7.9) | NA[b] | 6 (6.4) | 5 (11.1) | 6 (11.4) | 7 (8.7) | 2 (6.8) | 1 (5.4) | 3 (7.0) | 1 (9.3) |
| Non-hormonal medication | 2 (5.6) | 6 (4.3) | 0.77 (0.18-4.93) | 4 (3.6) | 3 (5.9) | 1 (1.7) | 3 (4.6) | 1 (3.3) | 1 (6.0) | 3 (7.1) | 0 (0) |
| Lubricants | 7 (18.5) | 41 (27.4) | 1.66 (0.72-4.33) | 27 (27.3) | 13 (28.0) | 12 (20.8) | 25 (32.7) | 8 (26.8) | 3 (19.7) | 11 (29.5) | 2 (23.5) |

| | | | | | | | | | | | |
|---|---|---|---|---|---|---|---|---|---|---|---|
| Surgery | 1 (3.0) | 4 (2.6) | 0.85 (0.13-12.90) | 2 (1.7) | 2 (4.4) | 2 (3.7) | 1 (1.2) | 0 (0) | 1 (6.6) | 2 (4.7) | 0 (0) |
| Psychotherapy | 1 (2.5) | 18 (11.8) | 5.30 (1.01-106.69) | 14 (14.4) | 3 (6.6) | 7 (12.6) | 7 (8.7) | 6 (19.9) | 0 (0) | 8 (19.9) | 0 (0) |
| Sex therapy | 1 (3.0) | 5 (3.4) | 1.13 (0.19-16.97) | 4 (4.1) | 1 (2.0) | 1 (1.7) | 0 (0) | 0 (0) | 0 (0) | 1 (2.4) | 0 (0) |
| Support groups | 0 (0) | 1 (0.7) | NA[b] | 1 (1.0) | 0 (0) | 1 (1.8) | 1 (1.4) | 0 (0) | 0 (0) | 0 (0) | 1 (12.5) |
| Relaxation techniques | 3 (8.1) | 18 (12.2) | 1.59 (0.51-6.81) | 15 (15.0) | 3 (6.6) | 7 (12.2) | 11 (14.5) | 6 (19.3) | 0 (0) | 4 (9.2) | 2 (25.4) |
| Pelvic floor training aids | 2 (5.1) | 16 (10.5) | 2.20 (0.58-14.62) | 11 (10.9) | 5 (10.0) | 4 (7.3) | 12 (15.4) | 8 (26.9) | 2 (13.3) | 6 (15.3) | 3 (35.7) |
| Dilators | 1 (3.0) | 3 (1.7) | 0.57 (0.07-9.17) | 2 (1.8) | 1 (1.7) | 2 (3.2) | 3 (3.4) | 1 (3.4) | 0 (0) | 1 (2.2) | 0 (0) |
| Physiotherapy | 0 (0) | 3 (1.9) | NA[b] | 2 (1.9) | 1 (2.0) | 1 (1.7) | 3 (3.7) | 2 (6.5) | 0 (0) | 2 (4.9) | 0 (0) |
| Biofeedback | 0 (0) | 0 (0) | NA[b] | 0 (0) | 0 (0) | 0 (0) | 0 (0) | 0 (0) | 0 (0) | 0 (0) | 0 (0) |
| TENS | 0 (0) | 2 (1.2) | NA[b] | 0 (0) | 1 (2.1) | 1 (1.7) | 1 (1.3) | 0 (0) | 0 (0) | 1 (2.0) | 0 (0) |
| Body therapies (eg, massage, osteopetrosis) | 2 (5.3) | 9 (5.8) | 1.10 (0.27-7.22) | 6 (5.6) | 3 (6.3) | 1 (2.1) | 5 (6.2) | 4 (13.5) | 1 (6.0) | 2 (4.8) | 0 (0) |
| Physical activity | 7 (18.7) | 19 (12.5) | 0.63 (0.25-1.70) | 16 (15.5) | 3 (6.5) | 5 (9.0) | 12 (15.4) | 5 (16.5) | 0 (0) | 3 (9.1) | 2 (25.4) |
| **Barriers, n (%)** | 65 | 197 | | 120 | 75 | 74 | 100 | 40 | 25 | 48 | 12 |
| **Lack of knowledge regarding...** | | | | | | | | | | | |
| contact persons | 19 (28.8) | 54 (27.5) | 0.94 (0.51 – 1.77) | 34 (28.5) | 19 (25.7) | 17 (22.7) | 31 (30.6) | 11 (26.1) | 8 (34.1) | 12 (25.4) | 3 (22.4) |
| available treatments | 12 (18.8) | 35 (17.6) | 0.92 (0.46 – 1.95) | 22 (18.2) | 13 (17.2) | 11 (14.9) | 18 (18.0) | 5 (12.1) | 5 (21.7) | 7 (14.8) | 2 (16.9) |
| effectiveness of treatments | 10 (15.4) | 32 (16.4) | 1.07 (0.51 – 2.41) | 23 (19.0) | 10 (12.7) | 14 (19.0) | 19 (18.9) | 8 (20.3) | 1 (4.2) | 9 (18.0) | 0 (0) |
| **Lack of services...** | | | | | | | | | | | |
| in the region or too long waiting times | 8 (12.3) | 32 (16.3) | 1.39 (0.63 – 3.36) | 22 (18.4) | 10 (13.3) | 11 (14.5) | 21 (21.5) | 4 (9.3) | 3 (12.4) | 7 (13.5) | 0 (0) |
| sensitive to mental health | 8 (11.7) | 42 (21.2) | 2.03 (0.93 – 4.95) | 37 (30.7) | 5 (6.5) | 12 (16.3) | 23 (22.7) | 17 (41.6) | 7 (26.8) | 11 (21.9) | 4 (35.2) |
| sensitive to physical illnesses | 2 (3.0) | 17 (8.5) | 2.99 (0.82 – 19.29) | 14 (11.4) | 3 (4.0) | 6 (7.9) | 8 (7.8) | 8 (18.7) | 5 (20.2) | 8 (16.3) | 1 (7.9) |
| sensitive to sexual orientation and gender identity | 4 (5.8) | 17 (8.8) | 1.58 (0.56 – 5.83) | 9 (7.9) | 7 (8.9) | 9 (12.0) | 7 (7.4) | 2 (4.4) | 3 (11.8) | 4 (9.2) | 1 (8.5) |
| sensitive to culture and religion | 6 (9.5) | 10 (4.8) | 0.48 (0.17 – 1.45) | 7 (5.5) | 3 (4.0) | 2 (2.9) | 4 (3.5) | 0 (0) | 2 (8.6) | 1 (2.2) | 0 (0) |
| **Intrapersonal...** | | | | | | | | | | | |
| Lack of time | 13 (20.1) | 21 (10.8) | 0.48 (0.23 – 1.04) | 11 (9.3) | 10 (13.5) | 4 (5.2) | 13 (13.0) | 5 (11.3) | 2 (6.8) | 5 (11.4) | 0 (0) |
| Shame | 28 (43.0) | 81 (41.1) | 0.93 (0.53 – 1.63) | 54 (45.3) | 27 (35.7) | 29 (38.6) | 41 (40.7) | 23 (55.8) | 5 (19.9) | 25 (50.7) | 2 (17.7) |
| Avoidance due to difficulty of topics | 20 (31.1) | 49 (25.1) | 0.74 (0.41 – 1.39) | 37 (31.1) | 12 (16.2) | 18 (24.5) | 26 (26.3) | 14 (34.7) | 4 (15.4) | 16 (32.3) | 3 (26.7) |
| Fear of being discovered | 17 (26.2) | 38 (19.1) | 0.67 (0.35 – 1.30) | 20 (16.6) | 18 (23.6) | 17 (23.5) | 24 (24.1) | 10 (24.9) | 6 (23.7) | 12 (24.2) | 5 (40.4) |
| Fear of not being taken seriously | 25 (38.3) | 56 (28.4) | 0.64 (0.36 – 1.15) | 35 (29.5) | 19 (24.8) | 17 (22.5) | 30 (30.2) | 12 (29.0) | 6 (26.4) | 17 (35.9) | 1 (8.1) |

Abbreviation: CHC, chronic health conditions. MH+, comorbid mental CHC. CHC MH-, CHC excluding MH. CV+, comorbid cardiovascular and metabolic CHC. GY+, comorbid gynecological CHC. IN+, comorbid infectious and inflammatory CHC. CA+, comorbid cancer CHC. PA+, comorbid pain-related CHC. NE+, comorbid neurological CHC. OR, odds ratio. NA, not applicable.

[a]Odds ratios are reported for the comparison of women with vs. without CHC.

[b]Odds ratios were not calculated because there was no event in one group.

**Table S12**. Healthcare preferences and needs of women with SD among CHC status, MH status, and comorbid CHC subgroups.

| | **no CHC (n=69)** | **CHC (n=207)** | **OR[a]** | **MH+ (n=127)** | **CHC MH- (n=78)** | **CV+ (n=75)** | **GY+ (n=104)** | **IN+ (n=43)** | **CA+ (n=26)** | **PA+ (n=51)** | **NE+ (n=12)** |
|---|---|---|---|---|---|---|---|---|---|---|---|
| **Information sources, n (%)** | 66 | 197 | | 120 | 75 | 74 | 99 | 42 | 23 | 48 | 12 |
| Internet | 23 (35.5) | 74 (37.4) | 1.09 (0.61-1.95) | 49 (40.8) | 24 (31.9) | 26 (34.6) | 36 (36.8) | 17 (40.0) | 7 (29.2) | 18 (37.4) | 6 (49.9) |
| Literature | 5 (8.2) | 34 (17.0) | 2.31 (0.96-6.66) | 21 (17.7) | 12 (16.4) | 10 (12.9) | 19 (19.2) | 7 (16.0) | 5 (21.7) | 5 (10.2) | 2 (18.3) |
| Partner(s) | 19 (28.1) | 45 (22.6) | 0.75 (0.40-1.42) | 28 (23.7) | 16 (21.5) | 14 (19.0) | 25 (25.0) | 11 (26.4) | 3 (12.9) | 12 (24.9) | 4 (32.4) |
| Friend(s) | 7 (10.6) | 36 (18.3) | 1.88 (0.84-4.78) | 24 (20.0) | 12 (16.2) | 17 (22.6) | 19 (19.1) | 6 (14.0) | 3 (15.1) | 7 (15.2) | 4 (33.0) |
| Peer network | 11 (17.0) | 33 (16.8) | 0.99 (0.48-2.14) | 26 (21.6) | 6 (8.6) | 11 (14.6) | 19 (19.1) | 6 (15.3) | 5 (20.3) | 9 (17.7) | 3 (27.2) |
| General practitioner | 5 (7.8) | 25 (12.7) | 1.72 (0.69-5.13) | 14 (11.8) | 10 (12.7) | 9 (12.7) | 13 (13.0) | 5 (12.0) | 2 (10.9) | 8 (16.4) | 1 (6.3) |
| Gynecologists | 42 (63.5) | 113 (57.4) | 0.77 (0.43-1.36) | 71 (59.1) | 42 (56.1) | 48 (65.4) | 57 (57.6) | 24 (57.5) | 12 (51.9) | 28 (57.7) | 6 (49.8) |
| Urologist | 1 (1.3) | 15 (7.7) | 6.10 (1.14-139.42) | 8 (6.6) | 7 (9.5) | 6 (8.4) | 8 (8.0) | 3 (7.2) | 2 (9.0) | 6 (12.8) | 0 (0) |
| **Dialog partners, n (%)** | 65 | 195 | | 117 | 76 | 74 | 98 | 41 | 24 | 46 | 12 |
| Partner(s) | 28 (43.5) | 69 (35.2) | 0.70 (0.40-1.25) | 37 (31.9) | 31 (41.1) | 25 (33.4) | 42 (42.7) | 15 (35.9) | 7 (28.3) | 18 (38.8) | 5 (42.6) |
| Family | 5 (7.3) | 15 (7.9) | 1.09 (0.40-3.52) | 9 (7.5) | 7 (8.8) | 7 (9.5) | 8 (7.7) | 3 (6.5) | 2 (7.5) | 4 (7.6) | 3 (22.7) |
| Friend(s) | 8 (12.6) | 40 (20.4) | 1.78 (0.83-4.24) | 29 (25.2) | 10 (13.5) | 13 (16.9) | 21 (21.4) | 8 (19.5) | 5 (19.0) | 11 (23.2) | 4 (30.7) |
| Peer network | 5 (7.2) | 16 (8.1) | 1.15 (0.42-3.73) | 13 (11.0) | 3 (3.9) | 5 (6.8) | 8 (8.0) | 2 (4.5) | 2 (7.6) | 3 (5.8) | 0 (0) |
| General practitioner | 4 (6.1) | 22 (11.0) | 1.91 (0.70-6.72) | 12 (10.2) | 10 (12.6) | 9 (12.0) | 9 (9.7) | 4 (9.0) | 3 (12.2) | 6 (12.6) | 1 (7.8) |
| Gynecologists | 37 (57.4) | 96 (49.1) | 0.71 (0.40-1.25) | 57 (48.3) | 37 (48.9) | 40 (53.6) | 46 (47.3) | 20 (47.1) | 10 (40.7) | 25 (54.6) | 4 (35.1) |
| Urologist | 3 (4.2) | 16 (8.3) | 2.05 (0.64-9.64) | 10 (8.2) | 7 (8.6) | 6 (8.3) | 9 (9.4) | 2 (4.6) | 3 (14.6) | 5 (10.6) | 0 (0) |
| Psychiatrist | 4 (6.1) | 18 (9.2) | 1.57 (0.56-5.59) | 14 (11.7) | 4 (5.5) | 6 (8.2) | 9 (9.5) | 2 (5.9) | 2 (8.5) | 6 (12.4) | 2 (18.3) |
| Other physician | 1 (1.4) | 7 (3.3) | 2.37 (0.39-49.90) | 4 (3.8) | 2 (2.7) | 1 (1.3) | 3 (2.9) | 3 (6.6) | 2 (8.3) | 2 (4.0) | 0 (0) |
| Psychotherapists | 7 (10.7) | 42 (21.7) | 2.32 (1.05-5.90) | 29 (24.8) | 13 (17.6) | 16 (21.3) | 26 (26.6) | 8 (18.4) | 4 (14.9) | 10 (22.4) | 2 (17.7) |
| **Treatment goals, n (%)** | 62 | 196 | | 121 | 73 | 73 | 99 | 41 | 24 | 49 | 12 |
| Increased QoL | 12 (18.9) | 39 (19.7) | 1.05 (0.52-2.23) | 26 (21.4) | 12 (16.3) | 15 (20.4) | 20 (20.2) | 10 (25.1) | 3 (12.5) | 9 (19.3) | 2 (16.6) |
| Health literacy | 4 (6.3) | 19 (9.5) | 1.58 (0.56-5.66) | 15 (12.2) | 4 (5.4) | 6 (8.6) | 10 (10.3) | 6 (14.2) | 4 (15.5) | 6 (11.8) | 1 (9.0) |
| Sexual satisfaction | 22 (35.1) | 75 (38.5) | 1.16 (0.65-2.12) | 46 (37.9) | 29 (39.4) | 33 (45.9) | 40 (40.7) | 16 (40.0) | 6 (25.3) | 20 (41.5) | 5 (43.1) |
| Relationship satisfaction | 21 (33.8) | 76 (38.7) | 1.24 (0.69-2.27) | 44 (36.6) | 31 (42.1) | 30 (40.9) | 38 (38.6) | 15 (37.4) | 5 (19.3) | 17 (35.7) | 1 (8.3) |
| Feeling safe and close | 7 (11.3) | 35 (17.9) | 1.72 (0.77-4.38) | 27 (22.7) | 8 (10.5) | 16 (22.1) | 15 (15.2) | 10 (24.3) | 2 (8.4) | 9 (18.0) | 1 (8.3) |
| Body and sexual self-esteem | 16 (25.6) | 82 (42.0) | 2.10 (1.14-4.05) | 57 (46.9) | 26 (35.2) | 28 (38.2) | 42 (42.7) | 19 (46.4) | 5 (19.2) | 21 (44.2) | 3 (27.2) |
| Less stress | 11 (17.7) | 42 (21.2) | 1.25 (0.62-2.69) | 28 (23.1) | 12 (17.0) | 11 (15.3) | 25 (25.6) | 8 (19.6) | 6 (27.0) | 9 (19.1) | 0 (0) |
| Sex for relaxation | 7 (11.2) | 46 (23.5) | 2.43 (1.10-6.14) | 31 (25.7) | 14 (19.3) | 14 (19.0) | 26 (26.6) | 10 (23.8) | 5 (21.7) | 12 (24.4) | 4 (30.3) |
| Increased desire | 20 (31.6) | 64 (32.8) | 1.06 (0.58-1.97) | 43 (35.8) | 20 (27.6) | 26 (35.0) | 38 (38.5) | 8 (18.3) | 8 (34.2) | 20 (41.5) | 4 (34.6) |
| Increased arousal | 15 (23.5) | 52 (26.4) | 1.17 (0.61-2.32) | 31 (25.6) | 21 (28.5) | 22 (30.4) | 27 (27.7) | 5 (12.3) | 8 (32.1) | 12 (25.7) | 3 (24.2) |
| Frequent orgasm | 15 (23.6) | 46 (23.6) | 1.00 (0.52-2.00) | 31 (25.9) | 15 (20.5) | 17 (22.6) | 22 (22.2) | 8 (20.5) | 3 (11.6) | 14 (29.4) | 3 (24.2) |
| Decreased pain | 13 (21.4) | 56 (28.3) | 1.45 (0.75-2.95) | 32 (26.4) | 24 (32.2) | 20 (27.4) | 32 (32.4) | 13 (32.6) | 5 (19.3) | 18 (37.3) | 5 (40.5) |
| More communication | 6 (9.4) | 16 (8.1) | 0.85 (0.33-2.48) | 9 (7.6) | 7 (9.2) | 5 (6.9) | 12 (12.6) | 2 (3.8) | 2 (7.6) | 2 (4.8) | 2 (16.8) |

| | | | | | | | | | | | |
|---|---|---|---|---|---|---|---|---|---|---|---|
| Increased knowledge | 2 (3.2) | 16 (8.3) | 2.71 (0.75-17.03) | 12 (9.8) | 5 (6.2) | 7 (9.1) | 7 (6.7) | 5 (12.1) | 2 (6.5) | 8 (17.0) | 1 (7.8) |
| Social participation | 2 (3.2) | 13 (6.5) | 2.06 (0.55-13.14) | 3 (2.7) | 9 (12.9) | 6 (7.5) | 8 (8.3) | 3 (6.9) | 4 (16.0) | 3 (5.4) | 1 (7.8) |
| **Preferred offerings, n (%)** | 68 | 202 | | 124 | 76 | 75 | 101 | 42 | 25 | 49 | 12 |
| Specialized clinics | 9 (13.1) | 46 (22.6) | 1.94 (0.93-4.45) | 31 (25.1) | 15 (19.2) | 14 (18.9) | 28 (27.4) | 13 (30.0) | 5 (22.1) | 13 (25.8) | 3 (23.6) |
| Drugs | 6 (9.2) | 48 (23.9) | 3.09 (1.37-8.15) | 25 (19.9) | 24 (31.0) | 18 (24.0) | 28 (27.5) | 5 (11.8) | 8 (31.1) | 13 (27.2) | 2 (17.5) |
| Surgery | 1 (1.5) | 14 (6.8) | 4.71 (0.95-77.33) | 8 (6.4) | 6 (7.6) | 3 (3.8) | 5 (4.8) | 3 (7.1) | 2 (8.9) | 6 (11.5) | 0 (0) |
| Psychotherapy | 14 (21.2) | 51 (25.5) | 1.27 (0.67-2.52) | 42 (34.0) | 9 (12.4) | 17 (22.3) | 34 (33.4) | 13 (30.0) | 1 (3.9) | 12 (25.2) | 1 (8.3) |
| Sex therapy | 10 (14.2) | 45 (22.1) | 1.71 (0.84-3.82) | 28 (22.6) | 15 (20.2) | 15 (19.3) | 21 (21.1) | 7 (17.6) | 2 (7.0) | 7 (15.0) | 3 (24.2) |
| Relaxation methods | 20 (29.3) | 67 (33.4) | 1.21 (0.67-2.22) | 43 (34.7) | 24 (32.0) | 22 (29.7) | 45 (44.8) | 16 (38.9) | 5 (19.4) | 18 (35.4) | 3 (26.2) |
| Peer counseling | 11 (15.5) | 42 (20.7) | 1.43 (0.71-3.11) | 26 (21.4) | 14 (19.0) | 14 (18.6) | 20 (19.8) | 8 (20.1) | 6 (24.5) | 8 (15.5) | 2 (17.3) |
| Physiotherapy | 10 (15.3) | 35 (17.4) | 1.16 (0.57-2.56) | 23 (18.4) | 12 (16.2) | 10 (12.9) | 22 (22.3) | 10 (23.8) | 3 (12.3) | 8 (17.2) | 2 (16.8) |
| Pelvic floor training aids | 13 (18.8) | 55 (27.3) | 1.62 (0.84-3.31) | 33 (26.4) | 22 (28.3) | 16 (20.9) | 36 (35.9) | 9 (20.4) | 5 (19.6) | 14 (28.6) | 3 (24.3) |
| Biofeedback | 7 (10.7) | 21 (10.2) | 0.95 (0.41-2.44) | 14 (11.5) | 6 (8.4) | 4 (5.0) | 14 (14.1) | 5 (11.7) | 2 (10.1) | 4 (8.9) | 1 (6.3) |
| Dilators | 1 (1.4) | 7 (3.5) | 2.57 (0.44-53.47) | 6 (4.7) | 1 (1.5) | 4 (5.4) | 3 (2.7) | 2 (4.8) | 1 (3.7) | 1 (1.8) | 0 (0) |
| Vibrators | 12 (17.5) | 36 (18.0) | 1.03 (0.51-2.18) | 27 (21.5) | 9 (11.7) | 10 (13.2) | 16 (15.8) | 5 (11.2) | 1 (4.1) | 8 (17.1) | 2 (15.9) |
| Body therapies (eg, osteopathm massage) | 10 (14.3) | 41 (20.1) | 1.51 (0.73-3.38) | 28 (22.4) | 13 (16.9) | 12 (15.5) | 21 (20.7) | 10 (23.5) | 3 (10.9) | 9 (18.2) | 2 (18.2) |
| Physical activity | 13 (18.4) | 42 (20.9) | 1.17 (0.60-2.42) | 28 (22.7) | 14 (18.5) | 10 (13.7) | 22 (22.0) | 10 (23.4) | 2 (8.5) | 11 (22.5) | 0 (0) |
| Nutrition | 3 (4.1) | 18 (8.8) | 2.25 (0.72-10.27) | 13 (10.4) | 5 (6.3) | 6 (8.5) | 12 (12.4) | 3 (6.7) | 1 (4.5) | 1 (1.7) | 1 (9.9) |
| **Favoured future developments, n (%)** | 59 | 179 | | 106 | 71 | 67 | 92 | 39 | 22 | 42 | 9 |
| Dugs | 6 (10.2) | 29 (16.2) | 1.71 (0.72-4.72) | 13 (12.5) | 16 (22.2) | 16 (24.4) | 21 (22.8) | 3 (8.0) | 5 (20.8) | 10 (24.5) | 1 (10.5) |
| Surgery | 59 | 179 | 1.60 (0.53-6.53) | 10 (9.2) | 6 (7.9) | 6 (8.6) | 8 (8.4) | 1 (2.5) | 2 (8.1) | 6 (13.8) | 1 (10.5) |
| Information offerings | 12 (20.3) | 60 (33.6) | 1.99 (1.01-4.14) | 33 (30.8) | 27 (37.8) | 21 (31.8) | 30 (32.6) | 12 (29.7) | 7 (32.9) | 15 (35.7) | 0 (0) |
| Digital offers | | | | | | | | | | | |
| app | 13 (21.9) | 43 (23.9) | 1.12 (0.57-2.32) | 24 (22.5) | 19 (26.8) | 15 (22.4) | 25 (27.7) | 10 (24.5) | 5 (24.5) | 13 (31.5) | 3 (30.1) |
| website | 9 (16.0) | 41 (23.2) | 1.59 (0.76-3.61) | 27 (25.4) | 15 (20.4) | 14 (21.0) | 24 (26.1) | 10 (26.1) | 5 (21.0) | 10 (23.0) | 1 (11.5) |
| home-aids | 10 (16.9) | 39 (21.7) | 1.37 (0.66-3.06) | 22 (20.6) | 16 (22.2) | 17 (25.4) | 20 (21.5) | 4 (9.8) | 6 (27.1) | 9 (20.5) | 1 (10.6) |
| with physical face-to-face treatments | 5 (9.2) | 20 (11.2) | 1.25 (0.49 – 3.69) | 12 (11.0) | 8 (11.7) | 3 (5.1) | 13 (14.2) | 5 (12.8) | 2 (8.6) | 6 (14.7) | 0 (0) |
| contact to experts | 11 (18.1) | 38 (21.0) | 1.20 (0.59-2.64) | 26 (24.7) | 12 (16.2) | 8 (12.6) | 22 (24.2) | 9 (22.0) | 7 (30.6) | 5 (11.7) | 2 (23.4) |
| Trainings | | | | | | | | | | | |
| of physicians | 10 (16.3) | 29 (16.3) | 1.00 (0.47-2.30) | 17 (16.3) | 12 (16.9) | 9 (13.6) | 14 (15.8) | 10 (24.5) | 2 (9.2) | 5 (12.1) | 2 (23.3) |
| of psychologists | 16 (27.7) | 32 (17.8) | 0.57 (0.29-1.13) | 25 (23.4) | 7 (10.1) | 12 (17.4) | 17 (18.2) | 9 (22.4) | 3 (12.9) | 7 (16.8) | 3 (34.9) |
| diversity and trauma[b] | 5 (8.6) | 29 (16.5) | 2.09 (0.84-6.25) | 18 (17.2) | 11 (15.9) | 9 (13.2) | 14 (15.1) | 11 (26.9) | 5 (20.9) | 5 (12.6) | 2 (19.7) |
| **Design of digital offers[c], Median (IQR)** | 64 | 195 | | 121 | 74 | 73 | 98 | 41 | 23 | 49 | 12 |
| Stand-alone | 7.0 (5.0-8.0) | 8.0 (5.0-9.0) | | 7.7 (5.3-9.0) | 7.8 (5.0-9.6) | 8.0 (5.0-10.0) | 7.0 (5.0-9.0) | 8.0 (6.0-9.4) | 8.0 (7.0-9.0) | 8.0 (6.0-10.0) | 8.0 (5.3-9.0) |

| | | | | | | | | | | | |
|---|---|---|---|---|---|---|---|---|---|---|---|
| Possibility to contact experts | 8.0 (6.0-10.0) | 8.0 (6.0-10.0) | | 8.0 (6.0-10.0) | 8.0 (6.6-9.7) | 8.0 (6.0-10.0) | 8.0 (6.0-10.0) | 8.0 (7.0-10.0) | 8.0 (7.0-9.0) | 9.0 (6.4-10.0) | 8.0 (6.0-8.6) |
| Integration of medical HCP | 7.0 (6.0-8.6) | 8.0 (5.5-9.0) | | 8.0 (5.0-9.0) | 8.0 (6.0-10.0) | 8.0 (6.0-10.0) | 8.0 (6.0-9.0) | 8.0 (6.1-10.0) | 7.2 (5.6-9.0) | 8.0 (5.0-10.0) | 7.0 (5.0-9.3) |
| Integration of psychological HCP | 7.0 (6.0-8.0) | 8.0 (6.0-9.0) | | 8.0 (6.0-9.0) | 7.0 (5.3-9.0) | 8.0 (6.0-10.0) | 7.2 (6.0-9.0) | 8.0 (6.0-9.0) | 7.7 (6.0-8.0) | 8.0 (5.0-9.0) | 6.1 (6.0-8.2) |
| Possibility to incorporate partners | 8.0 (6.0-9.7) | 8.0 (5.0 -10.0) | | 7.5 (5.0-9.0) | 8.0 (6.0-10.0) | 8.0 (5.0-10.0) | 7.9 (5.0-10.0) | 8.0 (5.6-9.8) | 8.0 (5.6-9.0) | 8.0 (5.0-10.0) | 8.0 (5.0-9.0) |
| Reimbursement | 9.0 (7.0-10.0) | 10.0 (7.6-10.0) | | 10.0 (8.0-10.0) | 9.0 (7.0-10.0) | 10.0 (6.9-10.0) | 9.0 (7.0-10.0) | 10.0 (8.0-10.0) | 9.0 (8.0-10.0) | 10.0 (7.1-10.0) | 10.0 (7.8-10.0) |
| **Expert contact, n (%)** | 61 | 183 | | 111 | 70 | 67 | 94 | 38 | 20 | 46 | 10 |
| Chat | 18 (29.1) | 56 (30.5) | 1.07 (0.58-2.04) | 40 (36.5) | 15 (20.7) | 20 (29.5) | 31 (33.3) | 12 (32.9) | 3 (16.0) | 12 (26.3) | 2 (17.3) |
| Video call | 13 (21.2) | 27 (14.9) | 0.65 (0.32-1.39) | 21 (19.0) | 5 (7.1) | 5 (8.0) | 10 (10.3) | 4 (9.9) | 3 (14.0) | 4 (9.8) | 0 (0) |
| E-Mail Feedback | 14 (23.3) | 46 (24.9) | 1.09 (0.56-2.20) | 26 (23.6) | 19 (27.7) | 16 (23.8) | 30 (32.1) | 9 (24.9) | 7 (32.4) | 15 (32.1) | 2 (19.1) |
| Contact to medical sex experts | 27 (45.2) | 107 (58.5) | 1.71 (0.96-3.06) | 69 (62.4) | 37 (52.8) | 37 (54.8) | 50 (53.5) | 19 (50.9) | 10 (48.1) | 27 (58.6) | 5 (53.4) |
| Contact to psychological sex experts | 21 (35.2) | 90 (49.1) | 1.77 (0.98-3.25) | 62 (56.3) | 27 (39.2) | 30 (44.4) | 49 (52.6) | 20 (53.3) | 7 (35.1) | 25 (55.5) | 3 (32.3) |
| **Amount willing to pay, n (%)** | 23 | 81 | | 50 | 29 | 34 | 31 | 15 | 7 | 17 | 3 |
| Nothing | 3 (13.4) | 13 (15.5) | | 9 (18.3) | 3 (9.3) | 4 (10.3) | 5 (17.4) | 3 (19.3) | 2 (26.5) | 4 (20.8) | 0 (0) |
| < €50 | 7 (29.6) | 19 (23.9) | | 10 (20.7) | 9 (31.0) | 13 (38.7) | 8 (26.1) | 5 (31.5) | 1 (15.6) | 6 (36.1) | 2 (67.9) |
| €51-100 | 6 (26.7) | 17 (21.2) | | 11 (21.9) | 6 (21.3) | 5 (13.5) | 4 (12.5) | 3 (18.0) | 0 (0) | 3 (17.1) | 1 (32.1) |
| €101-300 | 4 (16.5) | 16 (19.7) | | 10 (19.3) | 6 (21.7) | 6 (16.6) | 10 (34.0) | 3 (18.9) | 2 (26.2) | 3 (15.3) | 0 (0) |
| > €300 | 3 (13.7) | 16 (19.7) | | 10 (19.8) | 5 (16.7) | 7 (20.8) | 3 (10.0) | 2 (12.2) | 2 (31.7) | 2 (10.7) | 0 (0) |

Abbreviation: CHC, chronic health conditions. MH+, comorbid mental CHC. CHC MH-, CHC excluding MH. CV+, comorbid cardiovascular and metabolic CHC. GY+, comorbid gynecological CHC. IN+, comorbid infectious and inflammatory CHC. CA+, comorbid cancer CHC. PA+, comorbid pain-related CHC. NE+, comorbid neurological CHC. OR, odds ratio. HCP, Healthcare provider.

[a]Odds ratios are reported for the comparison of women with vs. without CHC.

[b]Sensitivity trainings for e.g., culture, religion, trauma, gender identity or sexual orientation.

[c]Numeric rating scale from 1-10.
